# Participatory research with carers: A systematic review and narrative synthesis

**DOI:** 10.1111/hex.13940

**Published:** 2023-12-21

**Authors:** Bryher Bowness, Claire Henderson, Samia C. Akhter Khan, Mia Akiba, Vanessa Lawrence

**Affiliations:** ^1^ King's College London, Institute of Psychiatry Psychology and Neuroscience London UK; ^2^ Department of Global Health and Social Medicine King's College London London UK

**Keywords:** caregiving, carers, codesign, community‐based participatory research, health and social care research, patient and public involvement, user involvement

## Abstract

**Introduction:**

As patient and public involvement (PPI) in research has become increasingly common, research‐based recommendations on its principles and impacts have been established. The specifics of conducting PPI are likely to differ when involving different groups. Family/informal carers for those with health conditions or disabilities have a lot to contribute to research, but instances of their involvement have yet to be reviewed.

**Objective:**

To systematically review and synthesize studies where family/informal carers have been involved in the research process, to develop an understanding of the benefits, barriers and facilitating factors.

**Methods:**

A search of five electronic databases was conducted using a combination of terms relating to carers, involvement and research. A grey literature search, expert consultation and hand‐searching were also used. Following screening, data extraction and quality assessment, a narrative synthesis incorporating thematic analysis was conducted.

**Findings:**

A total of 55 studies met the inclusion criteria, with diverse design and participatory approaches. Four themes were identified, relating to the outcomes, challenges, and practicalities of involving carers: *(re) building relationships with carers; carers as equals not afterthoughts; carers have unique experiences; carers create change*. Full involvement throughout the research was not always possible, due to barriers from the research world and responsibilities of the caring role. The literature demonstrated ways for carers to contribute in ways that suited them, maximizing their impact, while attending to relationships and power imbalances.

**Conclusion:**

By summarizing the reported instances of carer involvement in research, this review brings together different examples of how successful research partnerships can be built with carers, despite various challenges. Carers are a heterogeneous group, and participatory approaches should be tailored to specific situations. Wider understanding of the challenges of conducting empowering research with carers, and the resources required to address these, are needed.

**Patient and Public Involvement:**

The initial findings and themes were presented to a group of carers who had been involved in research and whose reflections informed the final synthesis.

## 1 INTRODUCTION

1.1

Recently, there has been an increased emphasis^1^ on ‘partnership and collaboration in the research process between researchers and those affected by the research’.^2^ ^,^^p.46^ Participatory research often acts as an umbrella term for varied methodologies^3^ aiming to cocreate knowledge.^4^ Approaches can be described on a continuum of levels of involvement,^5^ which vary in the distribution of power between researchers and participants at different stages in the research process.^3^ This can include public and patient involvement (PPI), or other methods such as participatory action research (PAR), where participants take ownership over the process of data collection and meaning making to influence social change.^4^ Participatory methodologies are often driven by emancipatory aims to empower marginalized groups, with an epistemology that places value on lived experience as a form of knowledge. Meanwhile, PPI is often motivated by improving the quality and relevance of research findings for consumers.^6^, ^7^ While it is important to recognise these distinct theoretical underpinnings^8^; it has been argued that these ideological and pragmatic rationales are complementary.^9^ Participatory research is not without its challenges, as it can require additional time, skills and funding, and must be conducted with consideration of power balances to avoid tokenism.^10^ The potential risks and costs of conducting research with the public should not be underestimated.^11^

The term ‘carer’ is frequently used in healthcare and research to describe a partner, family member, parent or friend who provides unpaid care for an individual with a health condition.^12^ Many do not define themselves as ‘carers’ and the term has been heavily criticized,^13^, ^14^, ^15^ although an agreed upon alternative has not yet been proposed.^16^ At a time when health and social care services are under pressure, carers are providing an increasing proportion of support.^17^ This can be rewarding for them,^18^ but it can also be hugely challenging, perhaps contributing to the higher rates of loneliness,^19^ poverty, social isolation, and physical health conditions amongst carers.^20^ Consequently, carers can bring useful insight into how to support the people they care for, as well as their own needs.^21^, ^22^ The contributions carers can make to research, improving research quality while empowering an societally undervalued group,^23^, ^24^ ‘are only beginning to be recognised’.^24^ ^,^^p.86^

There have been numerous reviews of participatory research and PPI in general, exploring its guiding principles,^25^ impact,^6^ challenges^10^ and mechanisms.^26^ However, there is a need for more research into the practicalities of conducting participatory research,^27^ which is context specific and will vary with different populations.^28^, ^29^, ^30^ Carers are often included in PPI groups, but reports rarely differentiate their roles from service users or the public,^31^ and guidance remains primarily focused on involving service users.^32^ However, there are potential differences in carers' motivations, barriers and benefits of involvement, necessitating consideration of a unique approach.^14^ There have been some scoping reviews exploring participatory methods with carers, but these have focused specifically on parents as coresearchers,^33^ carers of those using mental health services,^34^ or photovoice with carers.^35^ To our knowledge, this is the first systematic review across methods and contexts, only including instances where carers have been the majority of public collaborators in a research project.

Carers are a heterogeneous group,^36^ and there is a great diversity in the rationale and methods of participatory approaches. This review takes a broad approach, including different types of carers and participatory approaches, to synthesize general benefits and recommendations. Researchers can then adjust these for their specific context and the individuals involved. Hopefully, this will encourage further ethical and research‐based involvement of carers in future.

## 2 METHODS

The protocol for this systematic review was registered on Prospero [CRD42021288437] and reported with reference to the PRISMA Checklist^37^ (Supporting Information S6).

### 2.1 Objectives

The aim was to provide a comprehensive overview of participatory research conducted with carers and the approaches used to identify the benefits, barriers and facilitators.

### 2.2 Search strategy and study selection

Systematic searches were conducted from inception until December 7 2021, then updated on March 6 2023. A comprehensive search combined sets of terms relating to (1) carers, (2) collaboration and (3) research (see Supporting Information S1 for full search narrative). As recommended in other reviews of participatory research,^33^ this review included a wide search strategy, searching five databases (Ovid MEDLINE, Ovid Embase, PsycINFO, CINAHL and Web of Science), grey literature (National Institute of Health Research [NIHR], NHS Evidence and PCORI databases), back‐referencing, and approaching experts in the field. References were collated on Endnote then uploaded in Rayyan for screening. The first 10% of titles/abstracts were independently screened by two researchers (B. B., M. A.) with 91.6% agreement, and discrepancies were discussed with supervisors (V. L., C. H.), before B. B. screened the remaining titles/abstracts. This process was repeated for the full‐text screening (agreement 80%). Authors were contacted for missing eligibility information before exclusion. Once an example of carer involvement was identified as eligible, associated publications were collected for data extraction.

### 2.3 Eligibility criteria

In accordance with the commonly used definition of public involvement as conducting research *with* rather than *on* or *for*,^38^ we excluded studies where carers only contributed as passive participants to the data. To select only higher levels of involvement, one‐off consultations were excluded. Moreover, studies where carers were *only* involved in priority‐setting, guideline development or intervention codesign were excluded due to our focus on involvement in the research *process* (despite the important contributions they can make to these stages). Only studies where carers, defined as those informally supporting someone with any health difficulty or disability,^39^ were the *majority* of public contributors were included. Each instance had to have available description of the participatory approach, research process and results, although this could be reported in multiple publications (full eligibility criteria Table 1).

**Table 1:** Inclusion and exclusion criteria.

	Inclusion criteria	Exclusion criteria
Study design		Research exclusion Intervention design only Priority setting/policy design only
Population	Carers are the majority of public contributors Informal carers such as partners, parents, family members, kin, friends Supporting those with mental or physical health problems, addictions, disabilities, frailty Self‐defined or otherwise Caring experience may be historic	Studies where lay members are involved but the majority are not informal carers Parents of children without health conditions/disabilities Formal carers/professionals/volunteers
Intervention	Involvement in a stage of the research process Research topic, design, research oversight, data collection, intervention design or delivery, data analysis, data dissemination Participatory data collection methods^a^ Involvement through consultation/advisory panel, part of the research team, carer‐led research	Involvement exclusion Carers are only involved as participants Carers only attend engagement event Carers are only involved piloting material Single one‐off consultation only Member‐checking
Comparators	N/A	N/A
Setting	Health or social care research Must involve some form of data collection	Nonhealth research (e.g., anthropological studies, educational interventions, normal development studies) Service evaluation/delivery/improvement projects Commentaries/theoretical papers
Outcome	Completed empirical study with description of the context method results	Incomplete studies Conference abstracts without published reports
	Description of carer involvement in the research process with details of the participatory approaches benefits barriers facilitators	Studies where there is no details of the participatory approach or outcomes of this

^a^ Defined as any method where participants have ownership over the direction and process of data collection, and the meaning making. These can include social mapping, body‐mapping, theatre, photovoice.^40^

### 2.4 Data extraction

Characteristics of the studies (e.g., population, methods), details of carer involvement, and outcomes and practicalities of this involvement (extracted verbatim), from all primary papers and their associated references were extracted into an Excel spreadsheet (example in Supporting Information S2). The absence of details was recorded as ‘not reported’. For the first six studies, this was completed by two researchers independently (B. B., V. L.), discussed and clarified, before the table was applied to the remainder by B. B.

### 2.5 Quality appraisal

The Mixed‐Methods Appraisal Tool (MMAT)^41^ was used to assess the quality of the research. As advised by its authors Hong et al.,^41^ total scores were considered with regard to the importance of individual items in context to the study, to determine whether the research methods were ‘high/medium/low’ quality. Where a team of carers and researchers had conducted multiple studies, each was assessed separately, with reference to all available reports. Two researchers independently rated 10 studies (78.5% agreement), and discrepancies were discussed (B. B., S. A. K.), before B. B. assessed the remainder. These appraisal tools may not capture studies with ideological underpinnings, nor do they reflect the quality of participatory processes. There is no standardized tool to appraise participatory approaches, so the ‘Critical Appraisal Guidelines for assessing the quality and impact of user involvement in research’,^9^ which claims to apply to diverse rationales, was selected. This was applied to the primary paper describing the participatory approach. Again, scores were considered in the context of the importance of individual items, to inform an overall categorization of ‘high/medium/low’ for the quality of what was *reported* about the ways carers were involved.

### 2.6 Data analysis and synthesis

A narrative synthesis approach^42^ was selected, as it suits reviews with diverse studies, allowing both a description of the research field and exploration of the implementation considerations. The approach has four key elements; (1) developing a theory of how the intervention works, (2) preliminary synthesis of findings, (3) exploring relationships between studies and (4) assessing the robustness of the synthesis.

A multitude of theoretical frameworks for participatory research^30^ have been proposed, but given the diversity incorporated in this review, one single theory was not applicable. Data extraction was limited to and mapped onto the research questions, then data was analysed to iteratively develop general recommendations for carer involvement across contexts.

The preliminary synthesis incorporated summary tables of study characteristics, a thematic analysis across studies, and discussions of the findings with three carers who had extensive experience of PPI. For the thematic analysis, verbatim data on the outcomes and practicalities of the participatory approaches was collated on NViVo. Two researchers (B. B., V. L.) independently coded data line‐by‐line from two diverse studies. Then, following discussion and refinement of their codes, B. B. semantically coded the remaining data and began to group codes into descriptive themes. Through ongoing discussions with V. L. and frequently returning to the data, these themes were interpretively rearranged into key analytic themes. Summaries and suggested themes were also presented to a NIHR carers steering group. This group provided reflections on the research approach and whether the themes accurately captured their experiences and the issues they thought were important. Their feedback informed the emphasis given to certain subthemes and directed the researcher to compare studies in additional ways.

Instances of different participatory approaches were compared to explore potential contributors their success. Approaches were categorized according to the ‘Levels and Stages of Service User/Survivor Involvement’^43^ (where each stage of a project can be understood as demonstrating ‘No involvement’, ‘Consultation’, ‘Contribution’, ‘Collaboration’ or ‘Control’). Some discretion was needed in applying this tool to participatory methods such as photovoice (see Table 2 footnote). The carers from the NIHR steering group queried how the context of the publication of the papers may influence report content. This informed further comparisons, such as exploring differences for papers with carer coauthors.

**Table 2:** Summary of participatory approaches according to ‘The Levels and Stages’.^43^

	Frequency (*n* = 55)	
Highest level of involvement		
Consultation	16	
Contribution	6	Where carer involvement in the research stage is separate to and sometimes overruled by researcher involvement (e.g., in photovoice analysis).
Collaboration	24	
Control	9	
Stages of involvement	Frequency^a^	Explanation/examples.
Research topic	26	Including coapplicants on grants.
Design/outcome measures	38	
Oversight	30	For example, grant coapplicants, steering committee members, ongoing communication with stakeholders, leading the PPI contribution.
Data collection	18	Excluding photovoice, workshop attendance or piloting materials.
Analysis	50	Including photovoice.
Intervention	35	Did not count towards inclusion of the study, but characterised as a means of involvement in conjunction with research involvement Including recommendations for services developed, or further action groups initiated.
Dissemination • Coauthors	34 • 20	Only where acknowledged that atleast one of the authors has lived experience as a carer.
All stages • All stages but not data collection	10 • 6	Research topic, design, data collection, data analysis and dissemination.

Abbreviation: PPI, public and patient involvement.

^a^ Total is more than 55 because some studies applied multiple forms of involvement within their approach.

Finally, the quality of all the included studies, the robustness of this review method, and discussions of whether the review's findings corresponded to the experiences of carers from the NIHR steering, informed reflection on the credibility and trustworthiness of the narrative synthesis conclusions (explored in Discussion).

## 3 RESULTS

### 3.1 Search and screening results

**Figure 1:**
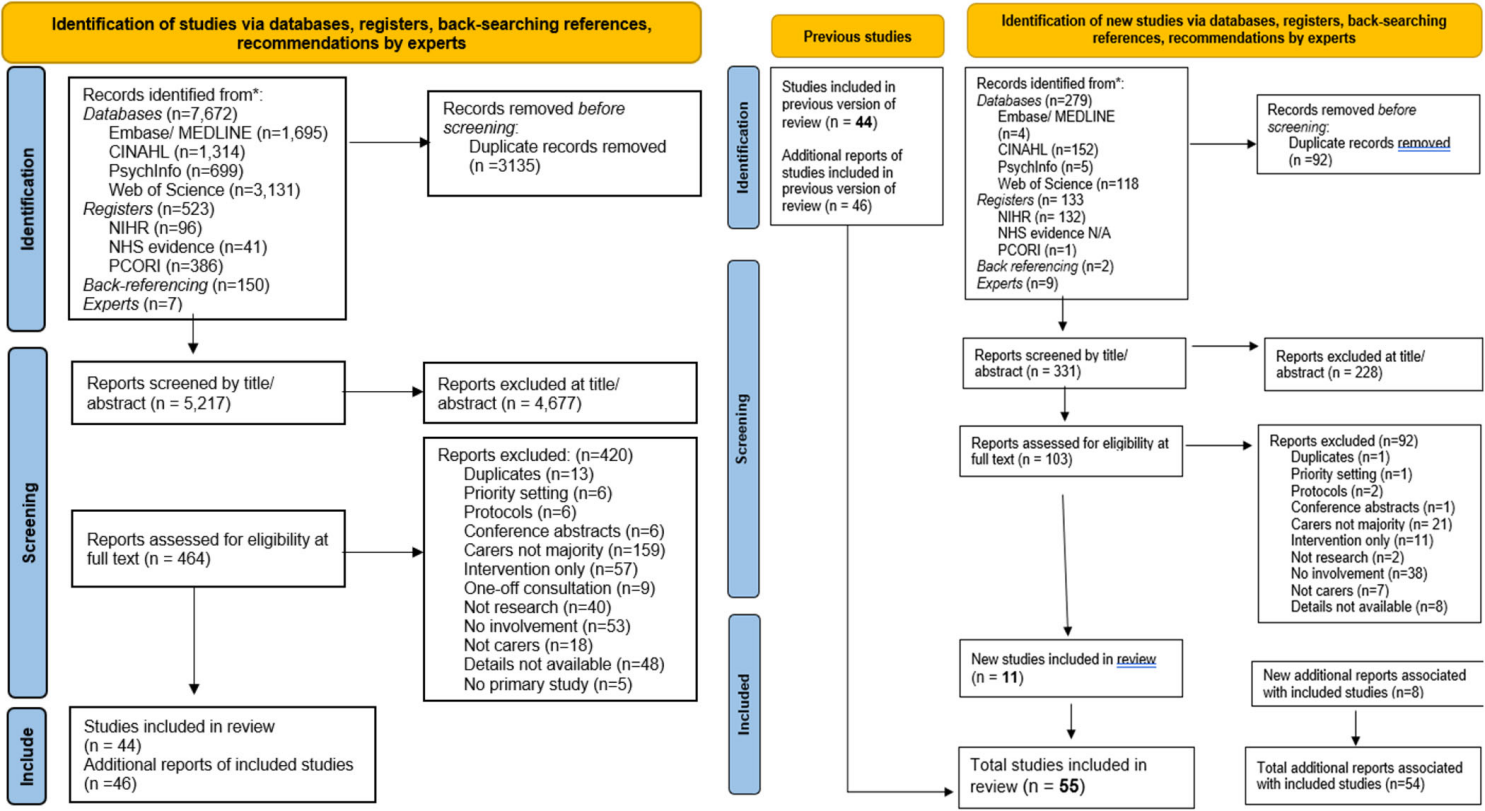
PRISMA flow diagram depicting references screened and reviewed, in initial and updated search (templates from ^44^).^37^

The initial search identified 8195 records; a further 157 were identified through backward reference searching and recommendations of experts (see Figure 1). Following deduplication, 5217 went through to the title/abstract screening. Common reasons for exclusion at this stage were that papers were only available as conference abstracts or protocols. A total of 464 papers were screened at full text, and 44 instances of carer involvement were included from this initial search. Updating the search identified a further 423 references, of which 11 met inclusion criteria, although one of these was a new publication associated with an already included reference.

Studies were frequently excluded because carers were not the majority of lay members involved (*n* = 159), only involved in intervention design (*n* = 57), or not at all (*n* = 53) (despite participatory claims, carers were only member‐checking results, piloting material, or being interviewed). In total, 55 instances of participatory research with carers were included, with data extracted from 109 papers, ranging from newsletters, full reports, and PhD theses.

### 3.2 Context, characteristics and quality of research methods and participatory approaches

See Table 3 for details of the included papers. Most studies were conducted in higher income countries; photovoice was conducted in South Korea,^45^ Peru,^46^ Malawi^47^, ^48^ and Kenya,^49^, ^50^, ^51^, ^52^ as well as with migrant and indigenous communities. Common areas of health were dementia, mental health and paediatrics. Studies demonstrated a wide variety of research designs (action‐research cycles, interviews, randomized control trials), of which 38 were purely qualitative, 14 mixed methods, eight quantitative methods, and two were reviews. Thirty‐one studies showed high‐quality research methods using the MMAT^41^ (out of 62 as some papers describing carer involvement produced multiple research projects) (see Supporting Information S3 for full scoring.

**Table 3:** Included references and summarized characteristics of the study and participatory approach.

Primary paper describing the participatory approach	Paper solely describing participatory approach	Co‐authored with carer/s?	Other associated references	Country/area of health	Aim/research methods	Description of involvement
Åkerman et al (2021)^54^/journal	No	No		Finland	Assess the feasibility of an Expert Caregiver intervention by co‐design and implementation/Mixed‐method pre‐post exploratory evaluation	Reference group + co‐design workshop + Carer‐led intervention
Argyle et al (2010)^55^/report	No	No		Ireland/Dementia	Explore carer experience of transition to care home/Explore carer experience of transition to care home/photovoice, interviews and focus groups	Participatory methods (photovoice)
Banfield et al (2018)^56^/journal	Yes	Yes	Morse et al (2019)^57^ journal Morse et al (2021)^58^ journal	Australia/Mental Health	Exploring views of ethics in mental health research^57^/interviews and focus groups Exploring views of ethics of involving carers^58^/interviews and focus groups	Carer researchers + Advisory group
Bates et al (2018a)^49^/journal	No	No	Bates et al (2018b)^48^ journal	Malawi/Palliative care	Explore patient and family experiences of care/photovoice	PAR Participatory methods (photovoice)
Bazzano et al (2015)^59^/journal	No	No		USA/Intellectual and Neurodevelopmental disabilities	Develop and evaluate stress reduction group for caregivers/Mixed‐method pre‐post evaluation	CBPR
Berry et al (2022)^60^/journal	Yes^61^	No	PPI Leaflet^61^	UK/Mental Health	Identifying barriers to psychological therapies in acute mental health settings and developing and testing an intervention/interviews	Advisory group + CDA
Bliss et al (2013)^62^/journal	No	No		USA/Dementia	Understand health literacy needs of carers relating to incontinence and develop educational material/focus groups, interviews, surveys	Advisory group (CBPR)
Cook et al (2019a)^63^/journal	Yes^64^	No	Cook et al (2019b)^64^ journal	UK/Intellectual Disabilities	Collaboratively develop and evaluate resilience building course for carers/action research	Project Team (Advisory group)+ PAR
Curtis et al (2018)^65^/report	No	No	Curtis et al (2018)^66^ journal	USA/Serious illness	Evaluation of a communication intervention to increase discussion of goals with patients/Multi‐center cluster randomized trial	Advisory group
Coupe & Mathieson (2020)^67^/journal	Yes	No	Mathieson^68^ (2019) thesis	UK/Oncology	Evaluate implementation of intervention for carers of cancer in community nursing/qualitative case study observation, interview, focus groups	Advisors (‘research buddies‘)
Deb & Limbu (2022)^69^/journal	No	No	Deb & Limbu (2021)^70^ journal	UK and Autralia/Intellectual and Neurodevelopmental Disability	Co‐designing and evaluate a training programme to reduce over‐medication of those with/EBCD and mixed methods field testing	Co‐applicants + Programme Development Group + Project Steering group + Co‐design workshop
Devlin et al (2022)^71^/journal	NO	No		Australia/Tuberculosis	Developing equitable care amongst Aboriginal peoples/Action research	PAR
dosReis et al (2019)^72^/report	No	Yes	Castillo et al (2018)^73^ journal Castillo et al (2016)^74^ journal	USA/Developmental disorder and co‐morbid psychiatric disorders	Design and evaluate decision‐making tool for ‘surrogates’/Mixed‐methods cross‐sectional (interviews, focus groups, discrete choice experiment, quantitative surveys)	Advisory group + Co‐researchers
Elliot (2013)^75^/thesis	No	No		USA/Young Carers	Participatory exploration of youth caregiver experiences and services to support them/mixed methods case study (document review, focus groups, and participatory arts‐based methods (body‐mapping, digital storytelling)	Participatory methods Co‐researchers CDA
Foster & Young (2015)^76^/journal	Yes	Yes	Modi et al (2019)^77^ ch8 report	UK/Neonatal care	Exploring parental attitudes to data sharing/quantitative survey	Co‐researcher + Advisory Group
Garner & Faucher (2014)^78^/journal	No	No		USA/Frailty	To explore challenges and support for carers/photovoice	CBPR Participatory methods (photovoice)
Giebel et al (2019)^79^/journal	No	Yes	Dalgarno et al (2021)^80^ journal	UK/Dementia	Explore carers experiences of home care and develop a dementia toolkit/qualitative survey	Advisory group + Steering committee + Data Management Committee + Co‐applicants + Co‐researcher
Grande et al (2023)^81^/journal	Yes	Yes	Bayliss et al (2023)^82^ journal Shield et al (2022)^83^ journal	UK/Palliative care	Systematic review of factors related to carers‘ mental health/qualitative meta‐synthesis	Carer co‐applicant + Advisory group + CDA
Hagen (1998)^84^/thesis	No	No	Gallagher & Hagen (1996)^85^ journal Hagen et al (1997)^86^ journal	Canada/Frailty	Establish and evaluate support group for carers/mixed methods focus groups and surveys	PAR Steering Committee
Hager et al (2021)^87^/journal	No	Yes		Sweden/Cystic Fybrosis	Propose and evaluate a conceptual model for patient‐controlled data/action research	Carer researcher
Hall et al (2023)^88^/report	No	No		UK/Elderly with loss of capacity (dementias)	Explore how professionals, older people and their families address challenges in managing money/document review and interviews	Advisory group
Hart & Neil (2021)^89^/journal	No	No		Canada/Down Syndrome (Intellectual Disability)	Explore support needs of carers/interviews and concept mapping	CDA
Hibberd et al (2009)^90^/journal	No	No		UK/Dementia	Explore how carers adapt their relationships/photovoice	Participatory methods (photovoice)
Kara (2016)^25^/journal	Yes	Yes		UK/Mental Health	Evaluate mental health carers’ research reference group/Qualitative and quantitative survey	Carer researchers + Advisory group
Kennedy et al (2011)^91^/report		Yes	Kennedy et al (2010)^92^ report	UK/Older adults with cancer (Oncology)	Exploring carers support needs and involving them in research/qualitative interviews focus groups	Advisory group
Kim et al (2017)^46^/journal	No	No		Korea/Paediatric Oncology	Exploring the experiences of carers of children cancer/photovoice	Participatory methods (photovoice)
Kowe et al (2021)^93^/journal	No	No	Kowe et al (2022)^94^ journal	Germany/Dementia	Exploring views on assistive technology for people with dementia in nursing homes/qualitative interviews	CDA
Lakhanpaul et al (2020)^95^/Journal	No	No	Lakhanpaul et al (2014)^96^ report	UK/Paediatric asthma	Develop intervention for South Asian children with asthma/Qualitative – Focus groups, interviews, workshops, Intervention Mapping Approach	CBPR + Co‐applicant + Carer co‐researcher +Advisory Group+ Co‐design workshops
Levy et al (2020a)^97^/Journal	No	No	Levy et al (2019)^98^ journal Levy et al (2020b)^99^ journal	USA/Paediatric palliative care	Understand experiences of carergivers^99^ Evaluate feasibility of online photovoice intervention for carers^98^ Evaluate effect of online photovoice intervention on carers’ wellbeing^97^	Participatory methods (photovoice)
Litherland et al (2018)^100^/Journal	Yes	Yes	O'Rourke et al (2021)^101^ journal Clare et al (2019)^102^ journal Clare et al (2022)^103^	UK/Dementia	Exploring the impact of COVID‐19 on people with dementia and their family^101^/qualitative interviews Develop a model that predicts capability to live well with dementia^102^, ^103^/longitudinal cohort study	Advisory group
Lobban et al (2020a) REACT^104^/Report	No	Yes	Lobban et al (2020b) REACT^105^ journal Robinson et al (2020)^106^ journal	UK/Bipolar and psychosis (Mental Health)	Evaluate online tool for carers (bipolar and psychosis)/Single blind quantitative RCT	Carer co‐researchers + Advisory Group + Steering Committee Management Group + Co‐applicant on grant +Codesign workshops + Carer led intervention
Lobban et al (2020c) IMPART^107^/report	No	Yes	Lobban et al (2020d) IMPART^108^ journal	UK/Bipolar and psychosis (Mental Health)	Identify factors affecting implementation of toolkit for carers/mixed method implementation study	Carer co‐researchers + Management Group + Steering Group + Carer‐led intervention
McCoy et al (2019)^109^/report	No	yes	e‐parent part I (Diller et al, 2017a)^110^ and II (Diller et al, 2017b)^111^ blogs	USA and Canada/Paediatric Cerebral Palsy	Create developmental trajectories for children with Cerebral Palsy/Prospective longitudinal cohort design	Advisory Group
Mitchell et al (2020)^112^/journal	Yes	Yes	Patchwood et al (2021)^113^ journal Darley et al (2021)^114^ journal	UK/Stoke	Development and evaluation of intervention to improve communication between professionals and carers/RCT^113^ Development and evaluation of intervention to improve communication between professionals and carers/Mixed method process evaluation^114^	Advisory Group+ Steering Committee
Morgan et al (2014)^115^/journal	Yes	Yes	Lanting et al 2011)^116^ journal O'Connell et al (2014)^117^ journal	Canada/Dementia	Exploring aboriginal experiences of aging and dementia/qualitative interviews Development and evaluation of a telehealth intervention for rural people with dementia/action research	CBPR Advisory group + CDA + carer‐led intervention
O'Sullivan & Hocking (2013)^118^/journal	No	No	O'Sullivan et al (2014a)^119^ journal O'Sullivan et al (2014b)^120^ journal O'Sullivan (2011)^121^ thesis	New Zealand/Dementia	Explore the daily activities of people with dementia/action research	CDA
Painter et al (2011)^122^/journal	No	No		USA/Paediatric mental health	Explore experiences of wraparound care’/qualitative interviews	Carer co‐researchers + Advisory Group + CDA
Parr et al (2021)^123^/journal	No	Yes		UK/Paediatric Neurological and Intellectual Disabilities	Exploring interventions available for parents to assist eating and drinking (child neuro‐disabilities)/mixed methods (focus groups, surveys, reviews, Delphi survey, stakeholder workshops)	Advisory group+ Co‐researchers + Co‐design workshops
Pletch et al (2015)^47^/journal	No	No		Peru/Paediatric clubfoot	Exploring experiences of caregivers using intervention for children with clubfoot/photovoice	CBPR (photovoice)
Postma et al (2015)^124^/journal	No	No		USA/Paediatric asthma	Exploring experience asthma management Mexican American parents/photovoice	PAR (photovoice)
Quinlan & Duggleby (2009)^125^/journal	No	No		Canada	Exploring participatory theatre with caregivers to elicit hope/action research (participatory theatre)	PAR Participatory methods
Ramfelt et al (2020)^126^ journal	No	No		Sweden/Paediatric diabetes	Develop and evaluate improvements support for family/EBCD	Co‐design workshops+ CDA
Rapaport et al (2018)^127^ journal	No	Yes	Livingston et al (2019)^128^ journal Alzheimer's Society^129^ (Bracken, 2021) newsletter	UK/Dementia	Feasibility and acceptability of intervention for caregiver strategies to help sleep disturbances for people with dementia/RCT	Co‐researchers + Co‐design workshops + Reference group
Repper et al (2007)^33^/book chapter	No	Yes	Repper et al (2008)^130^ report	UK/Mental health	Evaluate experiences of carers assessments/multi‐site case studies (document reviews, interviews)	Advisory group + Reference group + + Co‐researchers +CDA
Rising Together Action Group (2022)^131^/report	No	Yes	Blog 1 (Girdwood & Downs, *nd*)^132^ and Blog 2 (Girdwood & Robinson, nd)^133^	Australia/mental health	Explore the experiences of mental health carer lived experience workforce and create recommendations/qualitative survey, photovoice, focus groups	Co‐researchers+ Participatory methods (photovoice)
Schwarze et al (2020)^134^/report	No	No	Mason et al (2019)^135^ journal	USA/(older patients having surgery)	Evaluating intervention for patients making decisions about surgery/quantitative RCT	Advisory group
Skovdal et al (2009)^53^/journal	No	No	Skovdal & Andreouli (2011)^52^ journal Skovdal & Ogutu (2009)^50^ journal Skovdal (2011)^51^ report	Kenya/Young carers for HIV/AIDs	Explore experience young carers in Africa and develop self‐directed intervention (HIV/AIDs)/photovoice, arts‐based participatory methods, and action research	Participatory methods + Project management (intervention only)
Song et al (2020)^136^/report	No	No	Song et al (2019)^137^ journal	USA/Paediatric disabilities	Compare service use before and after policy change in care model/quasi‐experimental mixed methods	Advisory group
Virdun (2021)^138^/thesis	No	No	Virdun et al (2019)^139^ journal	Australia/Palliative care	Explore experience living with palliative care needs in acute care settings and improve systems/mixed sequential dominant design (systematic review, qualitative interviews, co‐design workshop)^138^, ^139^, ^140^, ^141^ Exploring preferences for engagement with service improvement in hospitals/qualitative interviews^142^	Advisory group + co‐design workshops + CDA
			Virdun et al (2020)^140^ journal			
			Virdun et al (2021)^141^ journal Virdun et al (2022)^142^ journal			
Walmsley & Mannan (2009)^143^/journal	No	No	Chadwick et al (2013)^144^ journal Mannan et al (2011)^145^ short report	Ireland/Intellectual Disabilities	Exploring the experience families of those with intellectual disabilities/focus groups	PAR Advisory group + co‐researchers
Walters et al (2023)^146^/journal	No	Yes		Australia/Mental Health	Explore engagement techniques used by family workers during COVID‐19/cooperative inquiry	Carer researchers (PAR)
Walters & Petrakis (2022)^147^/journal	No	No*		Australia/Mental Health	Explore the experiences of mental health carers during COVID‐19/rapid scoping review	Project Steering Group
Watson & Fox^148^ (2018)/journal	Yes	Yes	Watson (2016)^149^ thesis Fox et al (2019)^150^ journal Watson (2017)^151^ book chapter	Australia/Mental Health	Explore experiences of young carers/qualitative interviews	PAR
Williamson et al (2020a)^152^/journal	No	No	Williamson et al (2020b)^153^ journal	USA/Intellectual Disabilities	Explore Native American adults with intellectual disabilities abilities and their carers experience health/photovoice	PAR Participatory methods (photovoice) + advisory group
Yuwen, Duran & Tan (2021)^154^/journal	No	No		USA/Paediatric asthma	Describe and compare Latino and non‐Latino self‐care practices, needs and technology preferences when caring for children with asthma during co‐design workshops/descriptive qualitative observations	Advisory group+ co‐design workshops

Primary paper describing the participatory approach	Who was involved	Stages of involvement^44^	Highest level of involvement^44^	Involvement quality *(critical appraisal guidelines* ^9^)	Research methods quality (*MMAT* ^42^ *)*
Åkerman et al (2021)^54^/journal	6 carers, 4 professionals	Oversight Intervention	Consultation	Low	Medium
Argyle et al (2010)^55^/report	14 carers (majority women Spouse, siblings, adult children)	Analysis	Consultation	Low	Medium
Banfield et al (2018)^56^/journal	5 carer researchers + Advisory group – 4 carers, 5 consumers	All	Control	High	High, High
Bates et al (2018a)^49^/journal	Participants‐7 carers Other‐7 community member field workers, 2 nurses	Analyis Dissemination	Collaboration	Medium	High
Bazzano et al (2015)^59^/journal	Not reported	Research topic Oversight Design Analysis Intervention Dissemination	Control	Medium	Low
Berry et al (2022)^60^/journal	PPI group – 10 (majority carers)	Research topic Design Analysis Intervention Dissemination	Collaboration	Medium	Low
Bliss et al (2013)^62^/journal	Not reported	Design Analysis Intervention	Consultation	Low	Low
Cook et al (2019a)^63^/journal	Project Team – 3 carers, 1 psychologist + Participants– 18 carers (17 females, multiple caring responsibilities, 5 employed, 30‐70 yrs)	Design Analysis Intervention	Collaboration	Medium	Medium
Curtis et al (2018)^65^/report	Advisory group 20‐25 (most meetings attended predominantly carers)	Design Intervention Analysis	Consultation	Low	Low
Coupe & Mathieson (2020)^67^/journal	2 carers	Oversight Analysis Intervention	Consultation	High	High
Deb & Limbu (2022)^69^/journal	Co‐applicants – 2carers + Programme Development Group – 2carers and multiple other stakeholders + Steering Committee – 1carer + Workshop – 7carers	Research topic Oversight Design Analysis Intervention	Collaboration	Low	Low
Devlin et al (2022)^71^/journal	Father of barramany	Research topic Analysis Intervention Dissemination	Collaboration	High	Medium
dosReis et al (2019)^72^/report	Advisory group – 4 carers, 8 community leaders + Intervention co‐design workshop– 42 carers	All	Collaboration	Medium	High
Elliot (2013)^75^/thesis	Body‐mapping – 7 carers Project – 3 carers 3males, 4 females Ethnically diverse	Design Data Collection Analysis Dissemination	Collaboration	High	High
Foster & Young (2015)^76^/journal	1 Carer Researcher + Advisory Group ‐ 11 carers (10 mothers, 1 father, majority white English,, 1 South Asian, different socioeconomic)	Design Data collection Analsyis Dissemination	Control	High	Medium
Garner & Faucher (2014)^78^/journal	5 carers	Analysis Dissemination	Collaboration	Low	High
Giebel et al (2019)^79^/journal	Steering Committee ‐ 8‐10 carers (11‐15 total, people with dementia, public) Virtual advisory group‐ 20 carers + Co‐researcher + 2 carers co‐applicants	Research topic Design Oversight Analysis Intervention Dissemination	Collaboration	Medium	High
Grande et al (2023)^81^/journal	Carer co‐applicant + Advisory group – 5 carers	All	Collaboration	High	N/A
Hagen (1998)^84^/thesis	Steering committee –50% former carers (present carers, retirees, nurses, social workers, mental health workers, activity coordinators) Coordinator – carer	Design Oversight Analysis Intervention	Collaboration	Medium	Low
Hager et al (2021)^87^/journal	1 father	All	Control	Low	Low
Hall et al (2023)^88^/report	Advisory group – 5carers, one older person, 1 professional	Research topic Design Oversight Analysis Dissemination	Consultation	Low	Medium
Hart & Neil (2021)^89^/journal	1 carer (parent) Participants – 24 carers 20 mothers, 4 fathers, mixed urban vs rural, mixed education and income, mixed number of children	Design Analysis	Consultation	Low	Medium
Hibberd et al (2009)^90^/journal	Participants 9 carers	Analysis	Control	Medium	High
Kara (2016)^25^/journal	11 carers (Multiple ethnicities, men and women 20s‐70s, Caring experience 6months‐50yrs, Also service users	All	Control	Medium	Low
Kennedy et al (2011)^91^/report	6 older carers	Research topic Design Oversight Data collection Dissemination	Collaboration	High	Low
Kim et al (2017)^46^/journal	5 carers Mothers, children under 12 yrs, undergoing treatment, 33‐42 yrs	Research topic Data analysis Dissemination	Control	Medium	High
Kowe et al (2021)^93^/journal	6 carers mean age of 63 yrs (29‐79 yrs) 83.3% female	Analysis Intervention	Collaboration	High	High
Lakhanpaul et al (2020)^95^/Journal	MDT Advisory Board‐ 2 carers (also paediatrician, school nurses, service manager) 1 carer co‐researcher 118 participants IM workshops‐ 33carers, 23 children, 62 community members, 37 HCPs Majority South Asian mothers	All	Collaboration	High	High
Levy et al (2020a)^97^/Journal	Participants‐ 30carers 89% female, 83% white, 50% married	Analysis Intervention	Consultation	Low	Low, Low, High
Litherland et al (2018)^100^/Journal	5 carers, 3 people with dementia	Design Oversight Analysis Dissemination	Consultation	High	High, Medium, Medium,
Lobban et al (2020a) REACT^104^/Report	1 carer co‐investigator (Parent) + Codesign workshops ‐ 24 carers (Majority mothers 45 yrs + , 10 yrs+ caring experience, don't use computers Advisory Group – intermittent involvement 11 carers	Research topic Design Oversight Analysis Intervention Dissemination	Collaboration	Low	High
Lobban et al (2020c) IMPART^107^/report	1 carer on steering committee Others not reported	All	Collaboration	High	Low
McCoy et al (2019)^109^/report	7 carers (mothers, children 7‐20 yrs varied disability 5 white 2 black Geographically diverse)	Oversight Analysis Intervention Dissemination	Contribution	Medium	Medium
Mitchell et al (2020)^112^/journal	Steering Committee – 1carer Advisory Group – 10carers 7women 3men 34‐76 yrs, 8partners 2 parents 3‐5 yrs post stroke 1 stroke survivor and carer	Research topic Design Oversight Analysis Intervention Dissemination	Collaboration	High	High, Medium
Morgan et al (2014)^115^/journal	Advisory group – 27 carers (also staff, managers, organizations) Lanting et al (2011) – 4 carers grandmothers, 59‐73 yrs, range languages O'Connell et al (2014) – 11carers spouses, middle‐aged – 83 yrs, varied time since diagnosis	Design Oversight Data collection Analysis Intervention Dissemination	Collaboration	Medium	High, Low
O'Sullivan & Hocking (2013)^118^/journal	Participants –5 carers, 4 people with dementia 5couples, mix of genders	Analysis Intervention	Consultation	Low	High
Painter et al (2011)^122^/journal	15 carers (supported by professional evaluators)	Research topic Design Data collection Analysis	Control	Medium	Medium
Parr et al (2021)^123^/journal	2 carer co‐researchers Advisory group‐ 5 carers Stakeholder workshops – 15carers, 19 HCPs	Design Oversight Data collection Analysis Dissemination	Collaboration	Low	High
Pletch et al (2015)^47^/journal	Participants‐ 5 carers	Analysis Dissemination	Contribution	Low	High
Postma et al (2015)^124^/journal	Participants‐ 10/11 carers	Analysis Dissemination	Contribution	Medium	High
Quinlan & Duggleby (2009)^125^/journal	Participants‐ 8 carers 20s‐80s, children, spouses, grandchildren, recently bereaved	Dissemination	Contribution	High	High
Ramfelt et al (2020)^126^ journal	Workshops – 8‐16carers, 4adolescents, 4HCPs, 1 teacher 2men, 6women, children diagnosed 1 yr+	Analysis Intervention	Contribution	Low	High
Rapaport et al (2018)^127^ journal	Co‐researchers – 2carers Workshop – 4carers Reference group – 1carer	Design Oversight Intervention Dissemination	Collaboration	High	High
Repper et al (2007)^33^/book chapter	Advisory group – 2carers Sibling Co‐researchers‐ 2‐5 carers per site Majority mothers 40‐65 yrs many years caring	Design Oversight Data collection Intervention Analysis Dissemination	Collaboration	High	Medium
Rising Together Action Group (2022)^131^/report	Co‐researchers ‐ 1 lived experience community lead, 4 carer lived experience workers Photovoice participants ‐ 10 carer lived experience workers photovoice participants)	All	Collaboration	High	High
Schwarze et al (2020)^134^/report	Advisory groups – over half carers at each site (each group comprised 21‐27 total)	Design Oversight Analysis Intervention Dissemination	Consultation	Low	Low
Skovdal et al (2009)^53^/journal	48 young carers	Analysis Intervention	Contribution	Low	High
Song et al (2020)^136^/report	Advisory group – 7carers, 5 organisation reps	Research topic Design Analysis Dissemination	Consultation	Low	Medium
Virdun (2021)^138^/thesis	Advisory group – 7 bereaved carers, 2 patients, 2 cancer survivors Analysis – 5 bereaved carers, 1patient	Design Analysis Intervention	Consultation	Medium	High, High
Walmsley & Mannan (2009)^143^/journal	Advisory group – 4 carers + Co‐researchers – 5 carers (1father, 4mothers)	All	Collaboration	High	High
Walters et al (2023)^146^/journal	8 staff of lived experience mental health carer organization (4 project officers, 4 family carer advisers)	All	Control	High	HIgh
Walters & Petrakis (2022)^147^/journal	Project Steering Group – 19 carers, 1 consumer	Research topic Design Oversight Data collection Analysis	Consultation	Low	N/A
Watson & Fox^148^ (2018)/journal	12 young carers 6boys, 6 girls, 12‐17 yrs, European heritage	Research topic Design Analysis	Consultation	High	High
Williamson et al (2020a)^152^/journal	Advisory group – 4carers, 2HCPs, 1 Native American advisory Participants – 4carers, 4 with IDDUncle, grandmother, 2mothers, 38‐63 yrs	Research topic Design Oversight Analysis Dissemination	Collaboration	High	Medium
Yuwen, Duran & Tan (2021)^154^/journal	Parent advisory group (numbers not specified)	Research topic Design Oversight Intervention	Consultation	Low	High

Abbreviations = CBPR–Community Based Participatory Research, PAR–Participatory Action Research, PPI–patient and public involvement, EBCD – Experience‐Based Co‐Design, MMAT – Mixed Methods Appraisal Tool

**Figure 2:**
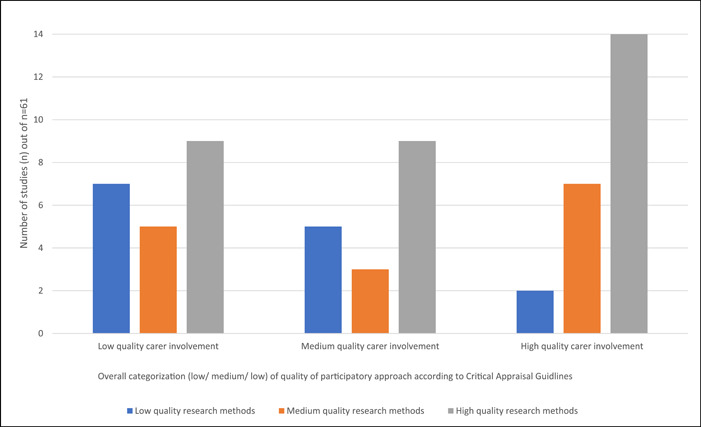
Distribution of quality of research methods using MMAT,^41^ according to quality of participatory approach using ‘Critical appraisal guidelines’.^9^ MMAT, Mixed Methods Appraisal Tool.

It was difficult to classify the different participatory approaches used, as authors used different terminology, and there was huge diversity within similar labels. Studies also combined means of involvement (e.g., workshops and advisory groups) (Figure 2).

**Figure 3:**
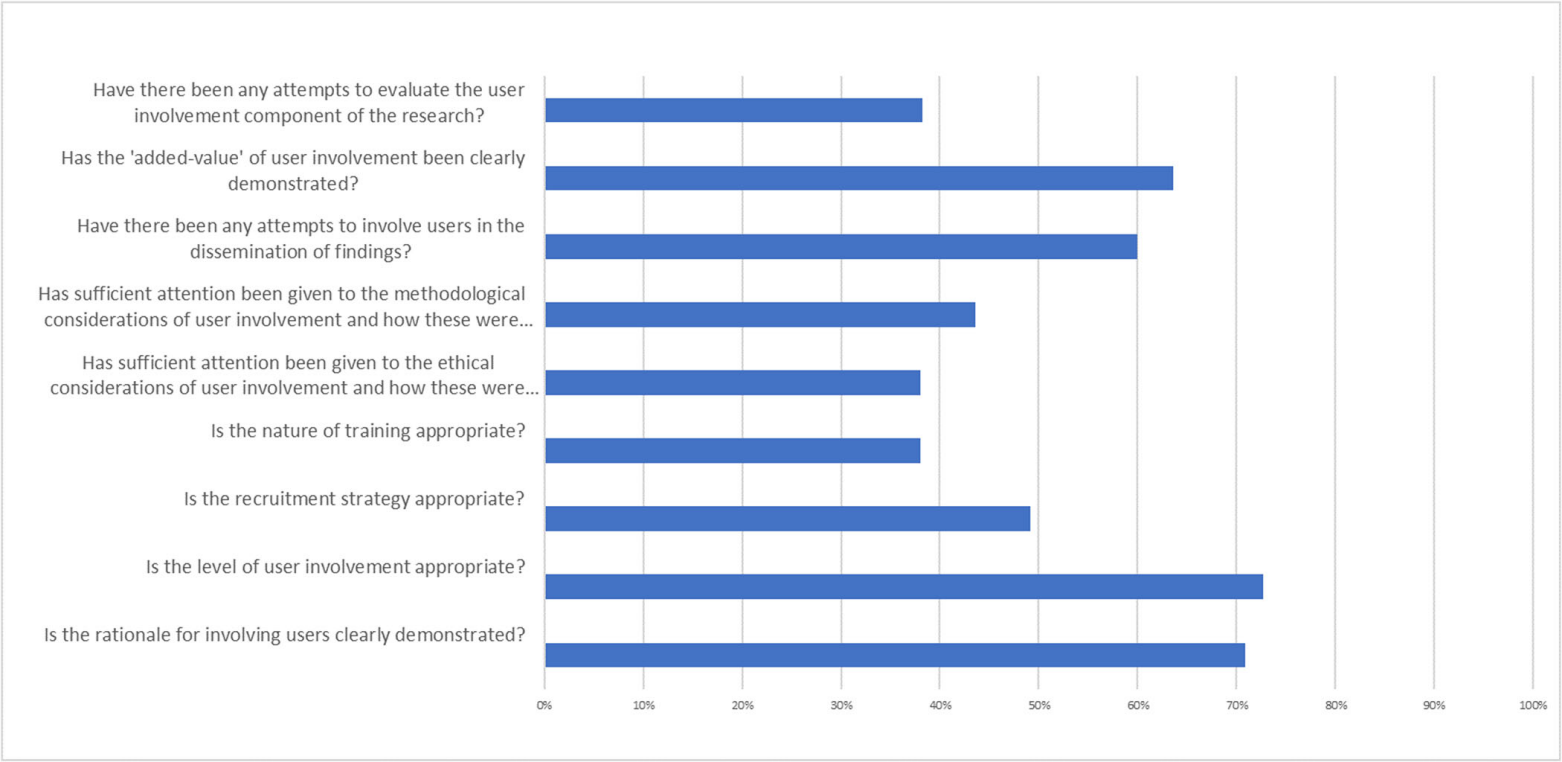
Percentage of participatory approaches evidencing each criteria of quality in ‘Critical appraisal guidelines’.^9^

See Table 2 for summary of the characteristics of participatory approaches. Most instances involved carers in the analysis of research, but carers also frequently contributed to design of the research study as well as the intervention (in cases where this was a component of the research). Involvement of carers in dissemination was also common through different means, with 20 instances of coauthorship. Almost half of studies involved carers collaboratively, and stages of research controlled by carers were also reported. Sixteen studies were judged to use only consultative methods (Figure 3).

High quality involvement was less common than high quality research methods (Figure 2). High quality research was most common amongst studies that also showed high quality participatory approach. Absence of appropriate training for carers, discussion of ethical issues relating to involvement, or evaluation of participatory approach, were amongst the common weaknesses (Figure 3). Many studies did not provide a rationale for using a participatory approach or show how carer involvement ‘added value’ (see Supporting Information S4 for full scoring).

### 3.3 Thematic analysis and lived experience reflections

Four overarching themes were identified relating to the benefits, barriers, and facilitating factors for the involvement; *(re)building relationships with carers, carers as equals not afterthoughts, carers have unique experiences*, and *carers create change* (see Supporting Information S5). Many of the subthemes resonated with the carers from the NIHR steering groups' experiences, but there were also elements they felt were missing from the literature or needed more emphasis.

#### 3.3.1 (Re)building relationships with carers

Conducting participatory research in relationship‐focused ways was particularly relevant to working with carers, to navigate the challenges of ‘perceived hierarchical differences between professionals and family carers, and family carer predominantly negative perceptions’,^62^ ^,^^p.805^ and bring together difference.^24^, ^145^ Authors described various facilitators of successful relationships. Additional time was needed to connect with carers as individuals,^118^ through frequent communication^74^, ^114^ and small, regular and informal meetings.^99^ Collaboratively establishing working agreements at the start^122^, ^124^ and constantly reviewing these together^55^ was recommended. ‘Researchers taking action when they said they would’^111^ ^,^^p.4^ or transparency when this was not possible,^126^ was helpful to build trust with carers, as was demonstrating long‐term commitment,^32^ continuity,^137^ and connecting with carers' communities^114^ during the research.

Trusting relationships were a key ingredient to successful participatory research, resulting in increased openness,^107^ ‘two‐way growth’,^55^ ^,^^p.1227^ and engagement of the wider community in research.^70^, ^71^ Reports of studies where carers were involved in fewer stages of the research tended to pay less attention to building relationships. Carers from the NIHR steering group pointed out that while trust and respect are essential for all public involvement, they have increased relevance to carers, who often have negative experiences of health services that can create barriers to research relationships.

#### 3.3.2 Carers as equals, not afterthoughts

The pressures and requirements of academia often limit the extent carers could be involved in the research. Barriers included complex bureaucratic structures, for example, the lengthy process of acquiring security checks required for carers' research access created long delays and additional work for carers and researchers.^32^ Similar frustrations were echoed in the NIHR steering group. These difficulties are likely to deter researchers and carers from involvement in certain stages of the research, particularly data collection. Resource constraints were another frequent explanation for making pragmatic adaptations to best practice participatory involvement.^94^, ^111^, ^152^ Decisions were frequently made before carers became involved, commonly due to the requirements of funders or ethics committees.^80^, ^147^ This may have led carers to feel as though their contributions were tokenistic^107^ or ‘like an add‐on’, (carer).^80^ ^,^^p.23^ This experience was also shared by carers from the NIHR steering group who felt their involvement was treated like a ‘they said, we did’ tick‐box exercise. The persistence of traditional hierarchical relations was also problematic; ‘I always felt a bit on the outside … as we did have a member(s) on the (RMG) who were ‘experts’ in PPI’, (carer).^80^ ^,^^p.23^ This may result in hesitancy amongst carers to contribute or offer criticism.^147^ Carers from the NIHR steering group suggested this risk was particularly relevant with carers, who may have experienced feeling diminished by healthcare professionals previously overruling or overlooking them. Finally, this review found that the majority of studies did not involve carers from diverse backgrounds.^78^, ^154^ Some studies (particularly participatory action research [PAR]) specifically focused on working with marginalized groups, but the measures to broaden access and inclusion proved costly^95^ and often required years of sustained community partnership.^47^

Papers described their efforts to work in more equitable ways with carers. Making the research accessible enabled meaningful contributions,^143^ e.g. presenting information in multiple formats and providing appropriate training.^107^ Researchers attended to power imbalances,^70^ for example, by disclosing their own personal experiences,^111^ or ensuring carers chaired/represented the majority in meetings.^64^ Establishing clear roles and expectations before involvement and being flexible in following carers' agendas^80^ gave carers more control during the process. Participatory methods for data collection were also effective in subverting traditional power imbalances, ‘as knowledge produced from the project was owned by the participants’.^152^ ^,^^p.4^ This also reduced the imposition of researcher values on data. When describing their experiences of involvement in research, the carers from the NIHR steering group noted that like with other marginalized groups, it was crucial to give space to be listened to, rather than dictating the agenda and expecting carers to talk about what was ‘relevant’, was crucial. Demonstrating that researchers valued the expertise of the carers^90^ through timely payment^99^ was key, particularly as much of the caring support they provide is unpaid. Recognition and appreciation was not only monetary; offering additional opportunities to carers (such as attending conferences) *‘*reinforced this sense of having something to offer’.^111^ ^,^^p.4^ Institutional support, such as supervisory and funding structures to provide adequate resources,^66^ having a whole team understanding of participatory research, and involvement of senior members, further lent importance to carers' roles in research,^111^ ^,^^p.7^ and also facilitated the process.

Papers dedicated solely to reflecting on carer involvement seemed more likely to describe themes of power dynamics or the challenges arising. While such papers have more space to discuss these issues, they were also more common when carer involvement had occurred in more stages of the research and through collaboration or control, and more frequently had carers as coauthors. When carers contributed to the writeup, papers showed more in‐depth exploration of all the themes (relationships, power, carer issues, and change). Carers from the NIHR steering group wondered whether there would be differences in the participatory approaches of newer studies, but the only apparent pattern seemed to be an increase in coauthorship in the last 10 years.

#### 3.3.3 Carers have unique experiences

A prominent theme in the literature was that the insight of the carers informed and improved the research. This was especially evident where carers contributed at the design and analysis stages.^32^ Early input ensured the relevance of the research,^94^ for example, carers identified which measures were important to them and their families.^126^ Studies claimed to have more ethical rigour,^57^ as carers highlighted potentially sensitive issues^75^ and suggested more appropriate support strategies,^138^ wording and methods.^65^, ^136^ Their understanding of the participants increased the efficacy of recruitment methods,^65^, ^66^, ^111^ and feasibility of participation to improve retention rates.^95^, ^105^ Carers helped to interpret ‘the meaning of that experience communicated by the carer’.^89^ ^,^^p.225^ Carers also identified key messages and additional implications for practice.^80^, ^113^, ^126^ An important caveat was that ‘there is not a single view held by carers but multiple perspectives’,^24^ ^,^^p.89^ which cannot be represented by one carer, something emphasised in consultation with the NIHR carers' steering group. Therefore, multiple carers should be involved.

Only one paper compared the quality of the data generated with carer involvement to that of researchers alone.^32^ This study found that participants actually spoke *less* openly when they were interviewed by carers as opposed to professionals. The authors suggested that the carers may lack confidence in interviewing, prompting them to question whether the intense resources required to provide adequate training could be more effectively spent at other stages of the research. However, other research studies claimed that participants who were carers felt safer to disclose more with carer researchers.^71^, ^143^ In these instances, carers cofacilitated interviews/focus groups with academics, highlighting the need for additional support during this stage and the benefits that can result from pairing both professional and caring experience in the research process.

The helpful insight carers brought was partly due to an increased awareness of some of the challenges carers may face, which also affected them during their involvement in research. For example, caring schedules,^147^ transport difficulties and feelings of guilt,^97^ hindered carer attendance of meetings.^83^ ‘Their full, complex and hectic lives meant they could only be involved in the research where it had direct meaning for them’.^62^ ^,^^p.810^ Strategies for managing some of these challenges included involving multiple carers for when some could not attend,^24^ or had to leave the project,^67^ and this also reduced the burden and likelihood of carer burnout.^83^ Given the fluctuations in the demands of caring, researchers extended the research schedule^97^ or created roles with more flexible commitment.^147^ Due to carers' limited time, it was recommended to ‘optimise the consumers input in a timely way’^138^ ^,^^p.649^ by focusing on tasks in which carers can efficiently contribute.^55^, ^75^

A carer from one of the studies stated that support for the carers involved in conducting the research should be as robust as that for the carers participating.^107^ The carers in the NIHR steering group also pointed out that it is often assumed that carers do not need as much emotional support as patients. Researchers may need to show sensitivity to the impact of changing family situations, for example one researcher^52^ created history profiles to avoid research demands during particularly vulnerable times. Involvement in interviewing other carers also presented challenges, triggering difficult emotions or dilemmas regarding disclosure.^32^ Many studies found that involving carers in groups created effective peer support systems for when issues during the research arose.^90^

Evident in the review was the need to involve carers in ways that suited them. Many carers found virtual methods helpful, such as online meetings or editing study material through emails.^78^ These allowed them to remain on hand if their family members needed them^74^ or fit involvement around their other responsibilities. However, sometimes virtual meetings were less effective in establishing relationships and felt less meaningful.^80^ ‘No one size fits all … so the optimum strategy was to employ a variety of methods’.^55^ ^,p.1226^ In the same way that carers are well‐placed to understand the needs and preferences of participants, researchers achieved convenient involvement by designing this with carers themselves. This was achieved either through consultations before the project^67^ or from the beginning, allowing the group to determine times, location, or length of meetings.^77^ ‘Having ongoing conversations with those contributing to the PPI about their experiences of the process and how they feel it could be improved enabled us to refine the process as it evolved and learn from our mistakes’ (academic researcher).^126^ ^,^^p.985^

#### 3.3.4 Carers create change

Few papers reported the carers' motivations to take on their roles in the research. Yet, a common theme was their desire to make a difference and to feel ‘like our family's experience could be of some help to other families in the future’ (carer).^110^

Delays and constraints on change due to limited capacity of the research project or health services were frustrating for carers,^66^, ^111^, ^114^ whose experience of living with conditions like dementia lent a sense of urgency.^99^ ^,^^p.1041^ Thus, researchers must update carers on the study progress.^136^ ‘Tangible evidence of their contributions, were key to their engagement’.^108^ ^,p.11^ This could be provided by an end product to carer involvement,^59^ such as an exhibition or new intervention. The carers in the NIHR steering group reflected on their disappointment when they felt researchers had just moved on after the project. Fortunately studies frequently described how the actual experience of working with the carers enthused and ‘prompted the researcher to go into action’.^117^ ^,^^p.25^ Participatory research influenced developments in healthcare to ensure ‘the concerns and issues of families at its centre’,^94^ ^,^^p.12^ with higher feasibility^58^ and sustainability.^114^ The support of carers ‘elevates respect for the project’ and ‘increased staff buy‐in’.^111^ ^,^^p.5^ This was effectively communicated to services when carers partook in dissemination and presentations.^74^ To effect change, services and key stakeholders must be included in the research process.^142^

Many carers felt empowered by their involvement in the research; ‘my experience counted for something… and it made me more confident’ (carer).^89^ ^,^^p.225^ During the process they formed networks to share knowledge and caring strategies.^90^, ^94^, ^123^ Participatory data collection methods, such as photovoice^47^ and bodymapping,^74^ provided opportunities to communicate a collective carers' voice. Groups of carers formed for the research project went on to ‘raise the profile of caregiving issues in their community, and to initiate efforts to address those issues’.^83^ ^,^^p.94^ Developing skills in research and advocacy,^123^ was ‘capacity building, enabling parents to begin to lead change … expand their collective power’.^142^ ^,^^p.274^

## 4 DISCUSSION

This review summarized peer‐reviewed publications and grey literature that described participatory approaches involving carers in the research process, and synthesized the reported methods, benefits, challenges and facilitators of these collaborations.

As with previous reviews of other public involvement,^155^ included studies used a range of designs (with the majority being qualitative), and involved carers across different stages of research. Participatory approaches were most frequently found in research with carers for those with dementia, intellectual disabilities, paediatrics or palliative care. Perhaps this is because carers are involved more when researchers deem patients to lack capacity, or because people are less likely to identify themselves as or be recognised as ‘carers’ in other areas of health.^156^

There were similarities in the benefits of involving carers in research to those established for participatory research approaches generally^157^; improved the quality of research, healthcare, and increased impact for those directly involved in the process and their wider communities. Characteristics of successful involvement were similar to the recommendations from guidance for wider PPI.^158^, ^159^ For example, authors attended to ‘principles’ (such as equality, transparency, and sensitivity) and ‘presence’ (by including multiple and diverse carers), echoing the 4PI National Involvement Standards.^159^ Best practice that was infrequent in research with carers was their involvement throughout the project. Often, researchers stated they were not able to work with carers as much as or in the ways they would have liked to, due to the contextual factors limiting opportunity for coproduction^160^ (discussed below). This review attempts to highlight some of the principles that may be especially important when working with carers, which can subsequently be prioritised if compromises are required in the extent or practicalities of the participatory approach.

Carers have limited time and busy lives. The burden of research for carers was ameliorated in ways like introducing flexibility and choice into the research schedule and carers' roles. However, these approaches were sometimes potentially less effective in building relationships with or empowering the carers involved. Perhaps the key recommendation was to allow carers themselves to design how they would like to be involved and to evaluate this with them throughout the process (although only a minority of studies did this). Another vital element to successfully involving carers was taking an action‐oriented approach and maximizing the impact carers' contributions have. Carers' commitment to improve care was reported repeatedly, which motivated them to overcome some of the challenges faced during their involvement.^83^ In previous research, carers reported reluctance to join PPI groups because they felt their previous research encounters had not resulted in meaningful change.^23^ Involving carers in projects with direct outcomes and communicating their impact to carers is essential.

This review found that carers commonly contributed in the design and interpretation, and dissemination stages of research, while involvement in data collection or topic identification was rarer. As with wider forms of participatory research,^161^, ^162^ the bureaucratic barriers, funding constraints, technical requirements and traditional assumptions and hierarchies detracted from full collaboration with carers. Involvement defined as ‘carer‐controlled’ was scarce, and some carers reported feeling their involvement was tokenistic. Attention to the impact wider systems have on power dynamics during carer involvement was crucial, especially because carers may come to the research feeling their voice has been unheard, undervalued and considered inferior by healthcare professionals in the past.^163^ To facilitate meaningful engagement, researchers made efforts to increase carers' agency and prioritized building relationships. Successful partnerships with carers through research initiated far‐reaching rewards, leading to ongoing reciprocal learning, trust, engagement of the wider community, and sustainable interventions.^55^, ^114^, ^142^ Unfortunately, most studies struggled to involve carers from diverse backgrounds, despite a recognition of the importance of intersectionality of caring experiences.^164^ Often researchers reported that this was due to the systematic barriers described above, intensified when attempting to include carers from marginalized groups. Wider institutional understanding and support is required to provide the financial resources and opportunities that communicate to carers the value of their contributions^90^, ^165^ and fulfil the emancipatory potential of participatory research.^163^, ^166^

This review found diverse and inconsistent descriptions of carer involvement, and a lack of clarity around terminology persists.^157^ This search found studies claiming to use participatory methods where carers had only piloted materials or participated in interviews, corroborating worries that terms such as coproduction are being misused and diluted.^167^ Many of the papers lacked an explicit rationale for participatory approaches, needed to guide decisions regarding the practicalities of involvement^166^ and ensure that carer input is maximally effective. The PAR studies in this review tended to follow a more consistent structure, with more explicit theoretical groundings and methodologies, which other involvement approaches could learn from.^168^ Without a stated rationale for what participatory approaches are aiming to achieve, it is difficult to determine their success.^169^, ^170^, ^171^ Although many studies found in this review reflected on their participatory approaches, very few conducted explicit evaluations with carers themselves. A further concern was the frequency of papers without any description of the challenges they encountered during involvement. Failures are often ‘brushed under the carpet’.^172^ ^,^^p.162^ but engagement with these issues is needed to improve practice. Clearer definitions, rationale and fuller reporting of carer involvement would better allow external scrutiny through systematic assessment of the quality of their participatory methods (a challenging aspect of this review). This would ensure researchers were held accountable,^78^ better credit carers for their contributions, and enable other researchers to form research‐based knowledge of ‘what works’ within different contexts.^173^ Recent years have seen the development of guidance for reporting the involvement process (GRIPP 2^174^), which may help to address this issue. Arguments for measuring the impact of public involvement, and tools for doing so,^175^ have been put forward as a means of providing research‐based justification for the significant time, resources and systematic changes that will be required to enable more equal research partnerships.^95^, ^171^, ^174^

The comparison stage of the narrative synthesis process.^42^ revealed the potential influence of the context of the report and who wrote it. The fact that the search found many instances of participatory approaches with carers that were only available as conference abstracts or protocols could suggest barriers for completing or publishing these. Peer‐reviewed journal articles either included in‐depth discussion of the themes or very little, likely due to differing focuses within restricted wordcounts. This lack of description of the involvement process was more common amongst studies that demonstrated higher quality research, perhaps because reporting traditional methodological rigour compromised space to reflect on the participatory process. Meanwhile, papers dedicated to discussing their participatory approach achieved more exploratory depth and provided more practical recommendations. Involving carers in the writeup is another way in which papers developed more reflective and insightful accounts of their participatory approaches. More papers reflecting on the participatory processes and increased coauthorship would be helpful in future to develop good practice for involving carers (Table 4).

### 4.1 Strengths and limitations

There was a generally high consensus in the issues and recommendations authors discussed about involving carers. The majority of studies were of high research quality, but this was not the case for the quality of the involvement, which limits the robustness of the recommendations drawn. However, studies rated as having higher quality involvement are likely to have had more influence over theme development,^176^ and indeed the studies with lower quality involvement were less likely to reflect on the challenges and practicalities of participatory research. Moreover, the carers who were consulted about the findings generally felt the review aligned with their views and experiences.

The challenges faced conducting this review were similar to those highlighted by previous reviewers of participatory research and PPI.^2^ Due to the lack of consistent definition and reporting of public involvement, it was difficult to design a search strategy that would identify all the relevant literature within a practical scope for screening. The likelihood that this review missed potentially eligible studies remained high, as multiple studies were found through backward citation searching and expert recommendations. Some bias may have been introduced during the selection process, for example, judgement was required to determine what was research ‘with’ carers.^38^, ^177^ There were additional challenges to determining whether carers were the majority (such as researchers not reporting the number of carers or their own identification as a carer, and the fluidity of carers' PPI membership).

The huge diversity of studies in this review is a strength, but created numerous challenges. A narrative synthesis approach incorporated difference through flexibility and multiple stages, but the process must be clearly documented.^42^ Theoretical frameworks can structure this interpretive process,^176^, ^178^ but the range of methodologies included in this review made it difficult to select models or quality assessment tools that suited all of these approaches. For example, categories in the ‘levels and stages of user involvement’^43^ was difficult to apply to photovoice, and the MMAT^41^ seemed to poorly capture quality in action research. A wide variety of carers, of different ages, cultures and supporting those with very different health conditions, were included (although diversity of carers within each study was lacking). However, it is important to acknowledge many others are providing care in circumstances not captured by the definition of carer used for this review.^179^ While this review attempts to draw out common themes, caution should be taken if generalizing these beyond the original context of each study (as with all conclusions from qualitative analysis^176^). Significant gaps in the literature remain where participatory approaches have not been applied, and learning from these instances would strengthen recommendations.

Finally, an important caveat to these recommendations is that systematic reviews summarize what the literature portrays, which may not reflect lived experiences of public involvement. Critical appraisal of the quality of the participatory approach for each paper also relied on author reporting, usually lacking the crucial perspective of the carers involved.^8^ Attempts were made to reveal the effects of reporting context by exploring differences in studies where carers were coauthors, and by incorporating the reflections of those with lived experience of involvement in research as carers to develop the final recommendations. The findings also resonated with studies that directly investigated carers' perceptions of research involvement.^180^, ^181^ However, the limited influence of carers during the research process of this review is a critical weakness.

## 5 CONCLUSION

This is the first review of participatory research and public involvement, specifically with carers, finding 55 examples. Carer involvement was successful across varied study designs and contexts, consistently adding value to healthcare research, while empowering carers and engaging their wider communities. Although many examples of strategies for overcoming the potential challenges of participatory approaches with carers are presented, achieving quality in public involvement depends on dynamic and relational factors. Thus, these recommendations Table 4 should be taken as general principles that should be applied according to individual contextual factors. There were many similarities found in the benefits, challenges and facilitating factors to those of general public involvement, but carer‐specific recommendations were also evident. Carers and researchers call for increased recognition of carers' value in research, and their distinct representation from patients and the public.^182^ Together with the NIHR's recently published resource of tips for researchers working with carers to conduct research,^183^ this review hopes to encourage and inform future carer involvement in research. However, wider institutional change is also required to maximise the full emancipatory potential of participatory approaches.

**Table 4:** Recommendations for facilitating effective carer involvement.

Benefits^a^	Strategy	Explanation/examples
Building trusting relationships	Engage with carers' communities and existing carer organisations.	*For example, involve members of these communities as facilitators, those communities, utilize existing engagement partnerships*.
	Maintain continuous communication—particularly relating to impact of carers' involvement and outcomes of their suggestions.	*For example, carer‐led newsletters throughout project, regular meetings, availability for ad‐hoc conversations, tailored communication methods*.
	Create informal engagement opportunities (small and face to face helps).	
Empowering participatory approach	Involve multiple carers.	*This can balance power dynamics in groups, and create ongoing peer support and advocacy groups*.
	Provide training and accessible methods of engagement.	*For example, provide information in advance and in different formats, avoiding jargon*.
	Communicating value of lived experience.	*For example, provide equitable renumeration, nonmonetary appreciation, acknowledgement/coauthorship*.
	Ensure institutional support.	*This will allow thorough supervision, planning, resources, involvement of senior academics in process*. *Create wider understanding amongst the whole team of the value of lived experience, wider understanding within governance structures of public involvement and a wider appreciation of time and funding requirements*.
	Create clear roles and expectations.	
Feasible participatory approach	Provide substantial and personalised practical and emotional support to carers involved.	
	Design the involvement strategy with carers.	*For example, ask an advisory group before, meeting at the start (when and how they would like to be involved, lengths of meetings, communication strategies)*, *or ask those involved at the start of the partnership*.
	Offer a multiplicity and variety of ways for carers to be involved.	*For example, online, arts‐based*.
	Use carers time efficiently.	
	Work flexibly and build in ongoing review of the process.	
Increasing impact of the project	Also involve stakeholders such as healthcare providers.	*For example, involve professionals too in the research,. create opportunities for carers to directly feedback/disseminate to stakeholders*. *This allows effective care relationships to develop*.
	Involve carers in creation of a tangible end product.	*For example, poster, film, intervention*.
	Support capacity building and future opportunities for carers.	*For example, conference attendance, ongoing advocacy roles*.
Encouraging future best practice	Consistent and transparent reporting of participatory approaches.	*Terminology for approach, structure of the report, include the reflections of carers involved, described the challenges and failures of participatory approaches*.

^a^ There are numerous other benefits to these approaches and strategies (not just one), as the positive effects are interrelated and amplifying.

## AUTHOR CONTRIBUTIONS

Bryher Bowness drafted the manuscript and Mia Akiba, Samia Akhter Khan, Vanessa Lawrence and Claire Henderson revised this. Bryher Bowness as responsible for the preparation of the manuscript. Bryher Bowness conceived the overall study. Bryher Bowness, Claire Henderson and Vanessa Lawrence developed the search strategy. Bryher Bowness conducted the search and contacted experts and authors for clarifications. Bryher Bowness and Mia Akiba conducted the screening in the initial review and Bryher Bowness conducted this in the update. Bryher Bowness and Samia Akhter Khan conducted the quality assessment in the initial review and Bryher Bowness conducted this in the update. Bryher Bowness and Vanessa Lawrence conducted the data extraction and Bryher Bowness conducted this in the update. Bryher Bowness conducted the analysis with wider input in the analysis design from Vanessa Lawrence and Claire Henderson.

## CONFLICT OF INTEREST STATEMENT

The authors declare no conflict of interest.

## Supporting information

Supporting information.

Supporting information.

Supporting information.

Supporting information.

Supporting information.

Supporting information.

## Data Availability

The data that supports the findings of this study are available in the Supporting Information material of this article.
