# Supplementary material for: Participatory research with carers: A systematic review and narrative synthesis
Source: Health Expect. 2023 Dec 21;27(1):e13940. doi: 10.1111/hex.13940 (PMC10734554; doi:10.1111/hex.13940)
Supplement: Supplementary file 1 — Supporting information. [file HEX-27-e13940-s001.docx]

**Supporting Information 1:** search narrative for systematic review

Search terms for systematic review

| Set 1 | Set 2 | Set 3 |
| --- | --- | --- |
| Community-Based Participatory Research* Community Participation  Patient Participation  *In the title…*  involv*  participat*  collaborat*  engag*  partnership*  representat*  “advisory panel”  co-produc*  emancipatory  co-research  consultative  co-design  “action research”  participatory  user-centred  user-led  expert*-by-experience | Carer*  Caregiver*  Care-giver*  Supporter*  Family care* Parent care* | Research  Study  Design  Method |

Combination of terms

((*exp.* Community-Base Participatory Research* / OR *exp.* Community Participation / * OR *exp.* Patient Participation* / AND (*exp.* Caregiver/

OR

(carer* OR caregiver* OR care-giver* OR supporter* OR family care* OR parent care*).tw)

AND

*In the title…*(involv* OR participat* OR collaborat* OR engag* OR partnership* OR representat* OR “advisory panel” OR co-produc* OR emancipatory OR co-research OR consultative OR co-design OR “action research” OR participatory OR user-centred OR user-led OR expert*-by-experience))

AND

*In the title…* (Research OR study OR design OR method*))

Sources

Ovid MEDLINE, Ovid Embase, PsycINFO, CINAHL and Web of Science.

Grey literature was searched through a database of NIHR funded studies involving carers since 2017, the first 2,500 studies searching ‘carers involvement research’ on NHS Evidence, and the results of searching ‘caregiver’ in the PCORI portfolio.

Search narrative

Following the full text screening of the references collected through the database and register search, the other search strategies were employed. This included approaching experts, and back searching the references of the included studies. These were then screened for eligibility. Identification of references for screening and inclusion continued following the initial search, due to this iterative process, as well as ongoing correspondence with experts. This was the case for the original search and the updated search.

Once a study was identified as meeting eligibility criteria, additional reports associated with this were identified through searching for the other publications of the included authors/ the study name on google scholar or within the study references. Where additional reports were found containing details of the participatory approach, they were included in the review for quality assessment and data extraction. Protocols were excluded (as they may not indicate eventual methods used). Of all the reports about a study, the report with the most detail regarding the participatory approach was identified as the primary paper.
