# Supplementary material for: Participatory research with carers: A systematic review and narrative synthesis
Source: Health Expect. 2023 Dec 21;27(1):e13940. doi: 10.1111/hex.13940 (PMC10734554; doi:10.1111/hex.13940)
Supplement: Supplementary file 2 — Supporting information. [file HEX-27-e13940-s003.docx]

**Supporting Information 2** data extraction table and example

| **Citation** | **Walmsley & Mannan (2009)** |
| --- | --- |
| **Type of paper** | Journal articles |
| **PPI reported separately?** | Yes |
| **Details of other publications?** | Chadwick et al (2012), Mannan et al (2011) and summary report |
| **Design** | Qualitative (focus groups) |
| **Aim** | To map the experiences of families of those with ID, whilst involving them in research |
| **Country** | Ireland |
| **Area of health** | Intellectual disabilities |
| **Sample** *(size / brief description of participant type)* | 70 families across Ireland caring for a child with an Intellectual Disability |
| ***Details of involvement*** | |
| **Rationale for involving lay members/ motivations of carers** | Walmsley & Mannan (2009) Empowering participants, increase relevance and rigor, reduce logistical problems, increase uptake of research results |
| **How authors describe level of involvement** | Participatory Action Research - collaboration among researchers and family members as coresearchers (Santelli et al, 1998; Turnbull et al, 1998). |
| **Details of methods of involvement** *(brief overview)* | Core family advisory committee and co-researchers who facilitated focus groups and participated in dissemination |
| **Highest 'level' involvement** (Sweeney & Morgan, 2009) | Collaboration |
| **Details of involvement in research topic** (Sweeney & Morgan, 2009) | Walmsley & Mannan (2009) Advisory group met with researchers to determine focus of research, |
| **Details of involvement in study design** (*detailed description of extent to which carers are involved in this process and specific contributions they made to study design)* | Walmsley & Mannan (2009) 1) Advisory group several meetings held to decide how to design the study- NIID staff presented different options to the family members. |
| **Details of involvement in outcome measures** (Sweeney & Morgan, 2009) | Walmsley & Mannan (2009) Questions were discussed with advisory group Chadwick et al (2012) topic guide questions developed prior to the involvement of the family co-researchers, howeverer they were reviewed and agreed by family co-researchers prior to data collection |
| **Details of involvement in study oversight/ management** *including involvement in recruitment, membership on a steering committee or project management group, grant applications and funding* | Helped with the recruitment of participants |
| **Details of involvement in data collection** *(detailed description of data collection methods which involved carers)* | Walmsley & Mannan (2009) Co-facilitated 7/9 focus groups - varying approaches (sometimes more of a resource, sometimes leading whole discussion. Chadwick et al (2012) Focus groups varied between 3-10 participants. Venues, dates and times were selected by the co-researchers. Conversational nature of focus groups allowed the family co-researchers who led the focus groups to ask additional probes. |
| **Details of involvement in data analysis** *(detailed description of data analysis methods which involved carers, with brief summary of researchers role in this analysis)* | Walmsley & Mannan (2009) "Throughout the analysis period, the lead researcher contacted co-researchers to member check of the analysis and interpretation," Findings presented to advisory group for "peer debriefing" Chadwick et al (2012) The extracted themes were taken back to the family co-researchers for discussion and further refinement; hence they were involved in the reviewing and confirming stage of the analysis process. Triangulation occurred - During this process the two researchers updated and discussed extracted themes and associated quotations with the other researcher and family co-researchers during meetings until agreement was reached about the final organizing themes. Member checks involved discussions with the family co-researchers and their involvement in the reviewing and confirming stage of the data analysis process. Subsequently, two member checking events were held in Dublin and Galway, at which the findings were summarized. At these, participants were asked if findings accurately represented their views. Family participants who attended indicated that organizing themes extracted did represent key issues in their lives. |
| **Details of involvement in intervention/ model/ service** *(including delivery and details of this if caregivers are involved in this) *not evaluation of the intervention - this goes in involvement in the research sections* | Walmsley & Mannan (2009) co-researchers commented on the potential creation of networks of parents across Ireland, an idea which also surfaced in three focus groups. practical mechanisms to achieve this have yet to be developed, |
| **Details of involvement in dissemination** | Walmsley & Mannan (2009) Three co-researchers and one advisor presented preliminary findings to Chief Executive Officers of service. |
| ***Who was involved?*** | |
| **Who was involved in the research** (*i.e. all those who aren't academics)? Total number of lay members involved? Number of carers?* | 4 parents advisory committee, 5 co-researchers |
| **Characteristics of carers involved?** | Walmsley & Mannan (2009) One father, four mothers. Chadwick et al (2012) "reflecting on this process the researchers involved felt that they had relatively mature, experienced, financially solvent, campaigning and confident family co-researchers working alongside them." p121 |
| **Recruitment of carers involved** *(with conducting research, not participants) Other organizations involved I recruitment process?* | Walmsley & Mannan (2009) advisory committee - all four parents had previous contact with NIID, co-facilitators - recruited following workshop mornings (Invitations were sent to 3000 families across Ireland through voluntary organisations including the 63 member organisations of the National Federation of Voluntary Bodies (NFOVB); Special Needs Active Parents Ireland (SNAP); 22Q11; Inclusion Ireland; and Down Syndrome Ireland. These organisations forwarded the letter to their members with a return address stamped envelope for families to indicate their willingness to participate in the study Chadwick et al (2012) Maximum variation sampling was used based on preliminary background information collected from the respondents to try and ensure that a range of families with differing characteristics were included in the study; specifically the views and experiences of families based in rural and urban locations, who had family members with intellectual disabilities of diffeing ages and with differing levels of support needs. |
| **Retention and engagement of co-researchers?** | 44 took part in training workshop, 10 expressed continued interest, 5 had availability to act as co-facilitators. Chadwick et al (2012) "most common reasons mentioned were that they did not have time available or mobility to travel" p121 |
| **Existing experience/ training of carers involved** *(not including delivery of the intervention, not including explaining the study)* | Walmsley & Mannan (2009) Training workshop piloted with 12parentsThe training workshop took 1.5 hours and covered the following activities which had been modified following feedback from the pilot. It provided an introduction to what a focus group is and what it was not. Participants were also provided an emphasis on establishing and agreeing upon ground rules for the group to observe during the discussion. The workshop also clearly outlined the role of the moderator, assistant moderator and note taker. The participants had an opportunity to practice these roles with the use of role plays. Chadwick et al (2012) The workshop offered a ‘hands-on’ opportunity to lead a subsequent focus group as a co-researcher and guidance on dealing with potentially difficult situations that may arise. Mannan et al (2011) THe training workshop was held on the day of the focus groups... consisted of written guidance, a presentation and oral instruction... this involved sharing information about setting up the room, the personnel required, setting a group culture, planning for and running the focus group. |
| **How is impact of involvement measured? How was the involvement evaluated?** *(focus only on evaluation of carer involvement) can include researcher reflection/ gaining informal feedback* | Interviews with authors 6months after Focus Group completion - structured questioons, thematic analysis 2) Augmented by review of focus group transcripts |
| ***Outcome of participatory method (verbatim for thematic analysis)*** | |
| **What impact did the method of involvement have on the study itself? What ways did the specific way in which the study involved carers benefit the quality of the study/ the results?** | Walmsley & Mannan (2009) Design "questions...Adaptations were made based upon their own lived experience...more open ended to capture the wide range of experiences","p37 Recruitment "Instrumental in informing the NIID researcher of the importance of selecting times and locations for focus groups which were convenient to parents"p37 Facilitating skills 'make a better participant when you have been through it' co-reaseher p275 "provided role models for other participants by being prepared to talk frankly about their experiences...helped to create a sesnse of safety" p273 CHadwick et al (2012) |
| **Benefits of method for researchers** | Not reported |
| **What impact did the method of involvement have for the carerss involved?** *Benefits for how PPI worked in the study?* | Walmsley & Mannan (2009) Empowerment - "potential benefits in terms of parents' empowerment"p274 "imparting skills to parents" p274,"capacity building, enabling parents to begin to lead change in their own localities" p274 begun to enable famillies to expand their collective power support " transmission of support and information between parents of different generations" p274 "you sit in a gruop of parents who know what you are talking about, it makes a huge difference" (co-researcher)p27 personal growth "opportunity to rethink his career options," p275 Chadwick et al (2012) empowered by the training, the networking, their participation in the focus groups and through their involvement in dissemination of the findings to services" p128 "many more family membersvolunteering to participate in training than expected," p130 "involvement in presenting the findings to local groups has also proved very effective in promoting carer-advocacy to families" p30 |
| **Benefits of method for effectiveness of intervention / improvement of health and social care services / impact on wider society / impact on cared-for individuals** | Not reported |
| **Disadvantages of the method/ barriers/ challenges/negative impacts?** *Were there any negative impacts of having carers involved in the research process (for either the individuals themselves, or for the research quality/ outcomes)? What were the difficulties carers or researchers faced when involving carers? Ethical issues?* | Walmsley & Mannan (2009) "Parents have too much to handle to get involved in leading groups," p274 2) practical mechanisms to achieve this have yet to be developed, and this presents a further challenge to the use of PAR p275 |
| **Ways used to overcome these** *Were there ways the researchers carried out the research which helped to overcome some of the difficulties of involving carers in research? Were there any adjustments to the study design in order to overcome difficulties or avoid negative impacts?* | Not reported |
| **Practicalities of carer researcher's participation -** *details of meetings? compensation? Communication methods? Support offered? How was PPI involvement structure decided?* | Not reported |
| **Recommendations for future** *Focus specifically on recommendations for involving carers in research/ recommendations from carers* | Walmsley & Mannan (2009) 1) "Professionals would be a valuable addition,"p275 "creating a dialogue between parents and service providers, possibly using the newly skilled co-researchers p275 2) "Planning for sustainability from the outset... would enhance the impact of the initative,"p275 |
| **Other** | Walmsley & Mannan (2009) "Without a control group it is hard to demonstrate conclusively that the presence of the co-researchers increased the relevance of the research" p274 |
