# Supplementary material for: Participatory research with carers: A systematic review and narrative synthesis
Source: Health Expect. 2023 Dec 21;27(1):e13940. doi: 10.1111/hex.13940 (PMC10734554; doi:10.1111/hex.13940)
Supplement: Supplementary file 3 — Supporting information. [file HEX-27-e13940-s006.docx]

**Supporting information 3 - Quality assessment of the research methods using MMAT** (Hong et al, 2018)

| ***Individual research studies (n=61)*** | ***Qualitative*** | | | | | ***Quantitative*** | | | | | ***Mixed methods*** | | | | | ***Overall*** |
| --- | --- | --- | --- | --- | --- | --- | --- | --- | --- | --- | --- | --- | --- | --- | --- | --- |
| *Randomized Control Trials* |  |  |  |  |  | **2.1** | **2.2** | **2.3** | **2.4** | **2.5** |  |  |  |  |  |  |
| Patchwood et al (2021)  *Associated primary paper Mitchell et al (2020)* |  |  |  |  |  | Y | Y | Y | Y | N |  |  |  |  |  | High |
| Livingston et al (2019)  *Associated primary paper Rapaport et al (2018)* |  |  |  |  |  | Y | Y | Y | Y | Y |  |  |  |  |  | High |
| Schwarze et al (2020) | Y | N | Y | Y | N | Y | Y | Y | Y | Y | N | N | N | N | N | Low |
| Lobban et al (2020) REACT | Y | Y | Y | Y | Y | Y | Y | N | Y | Y | N | N | Y | Y | Y | High |
| Curtis et al (2018) | Y | Y | Y | N | N | Y | N | N | Y | N | N | Y | N | Y | N | Low |
| *Non-randomized Control Trials* |  |  |  |  |  | **3.1** | **3.2** | **3.3** | **3.4** | **3.5** |  |  |  |  |  |  |
| Clare et al (2019)  *Associated primary paper Litherland et al (2018)* |  |  |  |  |  | Y | Y | N | N | Y |  |  |  |  |  | Medium |
| Clare et al (2020)  *Associated primary paper Litherland et al (2018)* |  |  |  |  |  | Y | Y | N | N | Y |  |  |  |  |  | Medium |
| Bazzano et al (2013) |  |  |  |  |  | N | Y | N | N | N |  |  |  |  |  | Low |
| McCoy et al (2019) |  |  |  |  |  | Y | Y | N | N | Y |  |  |  |  |  | Medium |
| Levy et al (2020a) |  |  |  |  |  | N | Y | Y | N | N |  |  |  |  |  | Low |
| Levy et al (2019)  *Associated primary paper Levy et al (2020a)* | Y | Y | N | Y | N | N | Y | N | N | Y | N | Y | N | N | N | Low |
| Lobban et al (2020) IMPART | Y | Y | Y | Y | Y | N | Y | N | N | N | Y | Y | Y | Y | N | Low |
| Hagen (1997) | Y | Y | Y | Y | Y | Y | N | N | N | N | Y | Y | Y | Y | N | Low |
| Song et al (2020) | Y | Y | N | Y | N | Y | Y | Y | N | Y | Y | Y | Y | N | N | Medium |
| Deb & Limbu (2021) | Y | Y | N | Y | N | N | Y | N | N | Y | Y | N | N | N | N | Low |
| Akerman et al (2021) | Y | N | Y | Y | N | Y | Y | Y | N | Y | Y | N | N | N | Y | Medium |
| *Descriptive* |  |  |  |  |  | **4.1** | **4.2** | **4.3** | **4.4** | **4.5** |  |  |  |  |  |  |
| Modi et al (2019)  *Associated primary paper Foster & Young (2018)* |  |  |  |  |  | Y | N | Y | N | N |  |  |  |  |  | Medium |
| Parr et al (2021) | Y | Y | Y | N | N | Y | N | Y | N | Y | Y | Y | Y | Y | N | High |
| Darley et al (2021)  *Associated primary paper Mitchell et al (2020)* | Y | Y | Y | Y | Y | N | N | Y | N | Y | Y | Y | Y | N | N | Medium |
| Hart & Neil (2021) | Y | Y | Y | Y | Y | Y | N | Y | N | Y | Y | Y | Y | N | N | Medium |
| Elliot (2013) | Y | Y | Y | Y | Y | Y | Y | Y | Y | Y | Y | N | Y | N | Y | High |
| dosReis et al (2019) | Y | Y | Y | Y | Y | Y | N | Y | N | Y | Y | Y | Y | Y | Y | High |
| *Qualitative* |  |  |  |  |  |  |  |  |  |  |  |  |  |  |  |  |
| Walters et al (2023) | Y | Y | N | Y | Y |  |  |  |  |  |  |  |  |  |  | High |
| Rising Together Action Group (2022) | Y | Y | Y | Y | Y |  |  |  |  |  |  |  |  |  |  | High |
| Chadwick et al (2012)  *Associated primary paper Walmsley & Mannan (2009)* | Y | Y | Y | Y | Y |  |  |  |  |  |  |  |  |  |  | High |
| O’rourke et al (2021)  *Associated primary paper Litherland et al (2018)* | Y | Y | Y | Y | N |  |  |  |  |  |  |  |  |  |  | High |
| Repper (2008)  *Associated primary paper Repper et al (2007)* | Y | Y | Y | Y | N |  |  |  |  |  |  |  |  |  |  | Medium |
| Williamson et al (2020) | Y | Y | N | N | Y |  |  |  |  |  |  |  |  |  |  | Medium |
| Quinlan & Duggleby (2009) | Y | Y | Y | Y | Y |  |  |  |  |  |  |  |  |  |  | High |
| Lakhanpaul et al (2014) | Y | Y | Y | Y | Y |  |  |  |  |  |  |  |  |  |  | High |
| Ramfelt et al (2020) | Y | Y | Y | Y | Y |  |  |  |  |  |  |  |  |  |  | High |
| Garner & Fauchner (2014) | Y | Y | Y | Y | Y |  |  |  |  |  |  |  |  |  |  | High |
| Postma et al (2015) | Y | Y | Y | Y | Y |  |  |  |  |  |  |  |  |  |  | High |
| Pletch et al (2015) | Y | Y | N | Y | Y |  |  |  |  |  |  |  |  |  |  | High |
| O’Sullivan & Hocking (2013) | Y | Y | Y | Y | Y |  |  |  |  |  |  |  |  |  |  | High |
| Dalgarno et al (2021)  *Associated primary paper Giebel et al (2019)* | Y | Y | Y | Y | N |  |  |  |  |  |  |  |  |  |  | High |
| Bliss et al (2013) | Y | Y | N | N | N |  |  |  |  |  |  |  |  |  |  | Low |
| Cook et al (2019) | Y | N | N | Y | Y |  |  |  |  |  |  |  |  |  |  | Medium |
| Painter et al (2011) | Y | N | Y | Y | N |  |  |  |  |  |  |  |  |  |  | Medium |
| Kara (2016) | Y | N | N | N | N |  |  |  |  |  |  |  |  |  |  | Low |
| Mathieson et al (2018)  *Associated primary paper Coupe & Mathieson (2020)* | Y | Y | Y | Y | Y |  |  |  |  |  |  |  |  |  |  | High |
| Lanting et al (2011)  *Associated primary paper Morgan et al (2014)* | Y | N | Y | Y | Y |  |  |  |  |  |  |  |  |  |  | High |
| O’Connell et al (2014)  *Associated primary paper Morgan et al (2014)* | N | Y | N | N | N |  |  |  |  |  |  |  |  |  |  | Low |
| Kennedy (2010)  *Associated primary paper Kennedy et al (2021)* | Y | N | N | Y | N |  |  |  |  |  |  |  |  |  |  | Low |
| Morse et al (2019)  *Associated primary paper Banfield et al (2021)* | Y | Y | Y | Y | Y |  |  |  |  |  |  |  |  |  |  | High |
| Morse et al (2021)  *Associated included reference Banfield et al (2021)* | Y | Y | N | Y | Y |  |  |  |  |  |  |  |  |  |  | High |
| Bates et al (2018) | Y | Y | N | Y | Y |  |  |  |  |  |  |  |  |  |  | High |
| Watson thesis  *Associated included reference Watson & Fox (2018)* | Y | Y | Y | Y | Y |  |  |  |  |  |  |  |  |  |  | High |
| Skovdal et al (2009) | Y | Y | Y | Y | Y |  |  |  |  |  |  |  |  |  |  | High |
| Argyle et al (2010) | Y | Y | N | Y | N |  |  |  |  |  |  |  |  |  |  | Medium |
| Hibberd et al (2009) | Y | Y | Y | Y | Y |  |  |  |  |  |  |  |  |  |  | High |
| Kim et al (2016) | Y | Y | Y | Y | N |  |  |  |  |  |  |  |  |  |  | High |
| Levy et al (2020b)  *Associated primary paper Levy et al (2020a)* | Y | Y | Y | Y | N |  |  |  |  |  |  |  |  |  |  | High |
| Kowe et al (2022) | N | Y | Y | Y | Y |  |  |  |  |  |  |  |  |  |  | High |
| Virdun et al (2022)  *Associated included reference Virdun (2019)* | Y | Y | Y | Y | N |  |  |  |  |  |  |  |  |  |  | High |
| Virdun et al (2021)  *Associated primary paper Virdun (2019)* | Y | Y | Y | Y | Y |  |  |  |  |  |  |  |  |  |  | High |
| Devlin et al (2021) | Y | Y | N | N | Y |  |  |  |  |  |  |  |  |  |  | Medium |
| Yuwen, Duran & Tan (2021) | N | Y | N | Y | N |  |  |  |  |  |  |  |  |  |  | High |
| Hall et al (2021) | Y | Y | N | Y | N |  |  |  |  |  |  |  |  |  |  | Medium |
| Berry et al (2022) | Y | N | N | Y | N |  |  |  |  |  |  |  |  |  |  | Low |
| Hager et al (2021) | N | Y | N | N | N |  |  |  |  |  |  |  |  |  |  | Low |
| ***Not applicable (systematic/ scoping reviews) (n=2)*** | | | | | | | | | | | | | | | | |
| Walters & Petrakis (2022) |  |  |  |  |  |  |  |  |  |  |  |  |  |  |  |  |
| Grande et al (2022) |  |  |  |  |  |  |  |  |  |  |  |  |  |  |  |  |

| ***Totals designs*** | *n=63* |
| --- | --- |
| Mixed methods | 14 |
| RCTS | 2 |
| Non-RCTS | 5 |
| Descriptive | 1 |
| Qualitative | 39 |
| Reviews | 2 |

| ***Totals overall MMAT ratings*** | *n=61* | *%* |
| --- | --- | --- |
| High | 31 | 50.8 |
| Medium | 15 | 24.6 |
| Low | 15 | 24.6 |

| *Total n=61* | | *Research methods quality ratings (MMAT)* | | |
| --- | --- | --- | --- | --- |
|  |  | Low | Medium | High |
| *Participatory approach quality ratings (Critical Appraisal Guidelines)* | Low | 7 | 5 | 9 |
|  | Medium | 5 | 3 | 9 |
|  | High | 2 | 7 | 14 |

Mixed Methods Appraisal Tool (MMAT), version 2018 (Hong et al, 2018)

| *Qualitative* |
| --- |
| 1.1 Is the qualitative approach appropriate to answer the research question? |
| 1.2 Are the qualitative data collection methods adequate to address the research question? |
| 1.3 Are the findings adequately derived from the data? |
| 1.4 Is the interpretation of results sufficiently substantiated by data? |
| 1.5 Is there coherence between qualitative data sources, collection, analysis and interpretations? |
|  |
| *Randomized controlled trials* |
| 2.1 Is randomization appropriately performed? |
| 2.2 Are the groups comparable at baseline? |
| 2.3 Are there complete outcome data? |
| 2.4 Are outcome assessors blinded to the intervention provided? |
| 2.5 Did the participants adhere to the assigned intervention? |
|  |
| *Non-randomized* |
| 3.1 Are the participants representative of the target population? |
| 3.2 Are measurements appropriate regarding both the outcome and intervention (or exposure)? |
| 3.3 Are there complete outcome data? |
| 3.4 Are the confounders accounted for in the design and analysis? |
| 3.5 During the study period, is the intervention administered (or exposure occurred) as intended? |
|  |
| *Quantitative descriptive* |
| 4.1 Is the sampling strategy relevant to address the research question? |
| 4.2 Is the sample representative of the target population? |
| 4.3 Are the measurements appropriate? |
| 4.4 Is the risk of nonresponse bias low? |
| 4.5 Is the statistical analysis appropriate to answer the research question? |
|  |
| *Mixed methods* |
| 5.1 Is there an adequate rationale for using a mixed methods design to address the research question? |
| 5.2 Are the different components of the study effectively integrated to answer the research question? |
| 5.3 Are the outputs of the integration of qualitative and quantitative components adequately interpreted? |
| 5.4 Are the divergences and inconsistencies between quantitative and qualitative results adequately addressed? |
| 5.5 Do the different components of the study adhere to the quality criteria of each tradition of the methods involved? |
