# Supplementary material for: Participatory research with carers: A systematic review and narrative synthesis
Source: Health Expect. 2023 Dec 21;27(1):e13940. doi: 10.1111/hex.13940 (PMC10734554; doi:10.1111/hex.13940)
Supplement: Supplementary file 4 — Supporting information. [file HEX-27-e13940-s004.docx]

**Supporting information 4** ratings according to application of the ‘Critical appraisal guidelines for assessing the quality and impact of user involvement in research’ (Wright et al, 2010)

| **Study*** | **1.** | **2.** | **3.** | **4.** | **5.** | **6** | **7** | **8** | **9** | **Overall** |
| --- | --- | --- | --- | --- | --- | --- | --- | --- | --- | --- |
| Rising Together Action Group (2022) | Y | Y | Y | Y | Y | Y | Y | Y | Y | High |
| Walters & Petrakis (2022) | Y | Y | N | N | N | N | Y | Y | N | Low |
| Walters et al (2023) | Y | Y | N | Y | N | Y | Y | Y | N | High |
| Walmsley & Mannan (2009) | Y | Y | Y | Y | N | N | Y | Y | Y | High |
| Litherland et al (2018) | N | Y | N | N | Y | Y | Y | Y | Y | High |
| Repper et al (2007) | Y | Y | Y | Y | Y | Y | Y | Y | N | High |
| Schwarze et al (2020) | N | Y | N | N | N | N | Y | N | N | Low |
| Lobban et al (2020) IMPART | Y | Y | N | Y | Y | Y | Y | N | Y | High |
| Foster & Young (2015) | Y | Y | Y | N | Y | Y | N | Y | Y | High |
| Hagen (1997) | Y | N | N | N | Y | Y | N | Y | Y | Medium |
| Williamson et al (2020) | Y | Y | Y | N | Y | Y | Y | Y | N | High |
| Quinlan & Duggleby (2009) | Y | N | Y | Y | Y | N | Y | Y | Y | High |
| Lakhanpaul et al (2014) | Y | Y | N | Y | N | Y | Y | Y | Y | High |
| Ramfelt et al (2020) | Y | Y | N | N | N | N | N | Y | N | Low |
| Hart & Neil (2021) | Y | N | Y | N | N | Y | N | N | N | Low |
| Hager et al (2021) | Y | Y | N | Y | N | N | Y | N | N | Low |
| Mitchell et al (2020) | Y | Y | Y | N | Y | N | Y | Y | Y | High |
| Garner & Fauchner (2014) | Y | N | Y | Y | N | N | Y | N | N | Low |
| Postma et al (2015) | Y | N | N | Y | N | Y | Y | Y | N | Medium |
| Pletch et al (2015) | N | N | N | Y | N | N | N | N | N | Low |
| Bazzano et al (2013) | Y | Y | N | N | N | N | Y | Y | N | Medium |
| Parr et al (2021) | N | Y | Y | N | N | N | Y | N | N | Low |
| O’Sullivan & Hocking (2013) | N | N | Y | N | N | N | N | N | N | Low |
| Giebel et al (2019) | Y | Y | N | N | N | N | Y | Y | Y | Medium |
| Bliss et al (2013) | N | N | Y | N | N | N | N | N | N | Low |
| Cook et al (2019) | Y | Y | N | N | N | Y | Y | Y | N | Medium |
| Painter et al (2011) | Y | Y | N | Y | N | N | Y | Y | N | Medium |
| Kara (2016) | Y | Y | Y | Y | N | N | Y | N | N | Medium |
| Lobban et al (2020) REACT | N | Y | Y | N | N | N | Y | N | N | Low |
| Curtis et al (2018) | N | Y | Y | N | N | N | N | Y | N | Low |
| dos Reis et al (2019) | N | Y | Y | Y | N | N | Y | Y | N | Medium |
| McCoy et al (2019) | N | N | Y | N | Y | N | Y | Y | N | Medium |
| Song et al (2020) | N | Y | Y | N | N | N | Y | N | N | Low |
| Coupe & Mathieson (2020) | Y | Y | Y | N | Y | Y | Y | Y | Y | High |
| Morgan et al (2014) | Y | Y | N | Y | N | Y | Y | Y | N | Medium |
| Kennedy et al (2011) | Y | Y | N | Y | Y | N | Y | Y | Y | High |
| Elliot (2013) | Y | Y | N | Y | Y | Y | Y | Y | Y | High |
| Banfield et al (2021) | Y | Y | N | Y | Y | N | Y | Y | Y | High |
| Bates et al (2018) | Y | Y | Y | N | Y | Y | Y | N | N | Medium |
| Virdun et al (2019) | Y | N | N | N | N | Y | N | Y | Y | Medium |
| Watson & Fox (2018) | Y | Y | N | Y | Y | Y | N | Y | Y | High |
| Skovdal et al (2009) | N | N | Y | Y | N | N | Y | N | N | Low |
| Argyle et al (2010) | Y | N | N | N | N | N | N | N | N | Low |
| Hibberd et al (2009) | Y | Y | N | N | Y | N | N | Y | N | Medium |
| Kim et al (2016) | Y | Y | Y | N | N | N | Y | N | N | Medium |
| Levy et al (2020) | N | N | N | N | N | N | Y | Y | Y | Low |
| Kowe et al (2021) | Y | Y | Y | N | Y | Y | N | Y | Y | High |
| Devlin et al (2022) | Y | Y | N | N | Y | Y | Y | Y | Y | High |
| Deb & Limbu (2022) | Y | Y | N | N | N | Y | N | N | N | Low |
| Akerman et al (2021) | N | N | Y | N | N | N | N | N | N | Low |
| Yuwen, Duran & Tan (2021) | Y | N | Y | N | N | N | N | N | N | Low |
| Hall et al (2023 | N | Y | N | N | N | N | N | N | N | Low |
| Rapaport et al (2018) | Y | Y | Y | N | Y | Y | Y | Y | Y | High |
| Berry et al (2022)/ TULIPS PPI leaflet | N | Y | N | N | N | Y | Y | Y | N | Medium |
| Grande et al (in press) | Y | Y | Y | Y | Y | Y | Y | Y | Y | High |
| **Totals out of n=55** | 39 | 40 | 27 | 21 | 21 | 24 | 38 | 35 | 21 | / |
| **As a %** | 70.9 | 72.7 | 49.1 | 38.2 | 38.2 | 43.6 | 60.1 | 63.6 | 38.2 | / |

* In the assessment of these criteria, the primary paper describing the participatory approach along with all its associated references were reviewed for reported evidence. The associated references are detailed in Table 4 in the main text.

| ***Totals overall participatory approach ratings*** | n=55 | As a % |
| --- | --- | --- |
| High | 20 | 36.4 |
| Medium | 15 | 27.3 |
| Low | 20 | 36.4 |

**Critical appraisal criteria for assessing the quality and impact of user involvement on health research (Wright et al, 2010)**

| 1. Is the rationale for involving users clearly demonstrated? |
| --- |
| 2. Is the level of user involvement appropriate? |
| 3. Is the recruitment strategy appropriate? |
| 4. Is the nature of training appropriate? |
| 5. Has sufficient attention been given to the ethical considerations of user involvement and how these were managed? |
| 6. Has sufficient attention been given to the methodological considerations of user involvement and how these were managed? |
| 7. Have there been any attempts to involve users in the dissemination of findings? |
| 8. Has the 'added-value' of user involvement been clearly demonstrated? |
| 9. Have there been any attempts to evaluate the user involvement component of the research? |
