# Supplementary material for: Participatory research with carers: A systematic review and narrative synthesis
Source: Health Expect. 2023 Dec 21;27(1):e13940. doi: 10.1111/hex.13940 (PMC10734554; doi:10.1111/hex.13940)
Supplement: Supplementary file 5 — Supporting information. [file HEX-27-e13940-s002.docx]

**Supporting information 5** thematic framework

| Subordinate themes | Example codes | Illustrative example quotes |  |
| --- | --- | --- | --- |
| **(re) Building relationships with carers**  *As with all collaborative research, building relationships are key to enabling effective involvement. Carers have often experienced negative encounters with healthcare professionals, increasing the importance of building open and trusting relationships through research, which in turn can lead to wider sustainable benefits.* | | | |
| The rewards of relationships | - Improved relationships with community and health services - Reciprocal learning - Enable openness - Increase commitment | *“expressed and demonstrated motivation to participate in health care and TB prevention activities, and family members established systems to support each other to participate,” Devlin et al, 2022, p729*  *“long-term commitment of the RUG was sustained by developing close working relationships and trust” Mitchel et al, 2020, p7*  *“meant that when it came time to have more difficult conversations, we had established relationships with each other and an established process for making change“ carer, RTAG, 2022, p19* |  |
| Relationships aren’t easy | - Preconceptions - Time is needed to establish community partnerships - Communication challenges - Emotionally draining - Challenges of bringing together difference - Relationships can create bias | *“perceived hierarchical differences between professionals and family carers, and family carer predominantly negative perceptions,” Cook et al, 2019, p805*  *“taking a significant amount of time to negotiate access, establish relationships and understand the context,” Coupe & Mathieson, 2020, p98*  *“those involved in co-production may not share the same kinds of knowledge or have the same understanding of what is or isn’t ethical” Kara, 2016, p87*  *“carers can find it difficult to say that they do not feel able to take on challenging work...it is important to spend time building relationships” Lobban et al, IMPART, 2020, Appendix 1, p125*  *“has the potential to inhibit the capacity to ‘stand back’ from,” Watson 2016, p95* |  |
| What goes into building relationships | - Being part of a team - Regular communication - Face-to-face contact - Openness - Trust - Long-term and continuity - Partnership working with other community organisations | *“we created a group identity: the group was named at our first meeting,” Litherland et al, 2018, p1038*  *“Small and informal helps here,” Litherland et al, 2018, p1041*  *“Trust came from the researchers taking action when they said they would,” Mitchell et al, 2020, p4*  *“Scaffolding co-production...* *These were initially instigated by the research leads , however, over time all group memers took up responsibility for creating a safe and supportive environment to work and learn in," RTAG 2022, p15*  *„methodology that was accessible to 'lived experience' researchers…bridge the gap between these two cultures [family carers and professionals]“ Walters et al (2023)* |  |
| **Carers as equals not afterthoughts**  *Carers often face additional societal disadvantages, yet their voice in healthcare and research remains rare. As with other marginalized groups, conducting public involvement in ways that value and promote carers’ voices, rather than perpetuates power differences, may be an important way of challenging wider inequality.* | | | |
| What stands in the way of equality | - Tokenism and power differences - Challenge relinquishing control - Research world, funding and bureaucracy - Those involved are not representative | *“I felt that the PPI involvement has been a bit sporadic and some of the researchers didn’t fully understand the benefits of involving carers or how to involve us,” carer quote, Lobban et al (2020) IMPART, p125*  *“frustrations were related to the fact that research had to be done in a certain way, within the confines of the protocol, and this could limit the changes they wished to make (e.g. changes to intervention tool could not be made due to copyright)" Mitchell et al, 2020, p6*  *“expectations of academia...having to make decisions before being allowed to work... submit four separate lengthy ethics applications," Watson & Fox, 2018, p480*  *“not being control in the traditional way creates anxiety,” facilitator quote, Cook et al, 2019, p809,*  *“should aim to capture the experiences of a wider range of PPCGs (paediatric palliative caregivers), including males and diverse/ racial ethnic backgrounds,” Levy et al, 2020, p369*  *“the gatekeeper role means the possible exclusion of those who were keen,” Watson 2016, p79* |  |
| Working towards equal partnerships | - Planning, resources and institutional support - Maximizing carer involvement and control - Mutually beneficial research that values carer experience - Attention to power imbalance in relationships – carer majorities build confidence - Clear roles and expectations - Accessible methods to enable meaningful contribution - Democratic discussions | *“to ensure carer voices were heard and valued the project focused on composition and chairing of the RAP meetings,” Grande et al, 2023, p10*  *“communicate their stories when and how they felt most comfortable,” Skovdal, 2009, p99*    *“effort is needed to understand the motivation for people to take part,” Robinson et al, 2020, p11*  *“designing safe spaces for reflexive practice was important given the perceived hierarchical differences between professionals and family carers,” Cook et al, 2019, p805*  *(analysis days) “these events were a key step in ensuring that participants felt fully part of the research process, rather than just providers of data,” Lobban et al, 2020, IMPART, p39*  *“opportunities for many of the RUG to get involved in teaching at the University, running workshops and presentations at national and international conferences also reinforced this sense of having something to offer and feeling valued” Mitchell et al, 2020, p4*  *“meetings are accessible – present them in an uncomplicated way, and often use diagrams and pictures, we usually have lots of questions,” Litherland et al, 2018, p1038*  *„early on in the study the acacdemis shared work that was in development rather than a finished project and the group were encouraged to openly criticque. This helped the group feel more comfortable putting forward their own ideas and being open to constructive criticism.“ RTAG, 2022, p17* |  |
| **Carers have unique experiences**  *Carers have valuable expertise to inform research, with insights of their own and those of their loved ones. However, their caring roles can also create challenges to their involvement, which must be facilitated to accommodate for these.* | | | |
| Carer experience benefits the study at all stages | - Early involvement - Understanding of participants creates relevant, ethical and effective research - Carers improve participant and researcher engagement - Lived experience enhances depth of understanding - Carers improve dissemination - Seek diverse carer perspectives | *“instrumental in informing the NIID researcher of the importance of selecting times and locations for the focus groups which were convenient to parents,” Walmsley & Mannan, 2009, p37*  *“impact was to establish trust with the community and study participants,” dos Reis et al, 2019, p13*    *“RAP members felt the perspective on caregiving from models was too linear, and did not reflect the dynamic nature of caregiving…further, they felt this perspective implied that negative mental health outcomes were inevitable,” Grande et al, 2023, p13-14*  *"representations of reality can be difficult to interpret without the meaning of that experience communicated (in this instance) by the carer," Hibberd et al, 2009, p225*  *“a huge leverage that the carers' voice has...people really listen if the carers say this is really important. It just seems to elevate people's respect for the project," researcher, Mitchell et al, 2020, p5*  *“there is not a single view held by carers but multiple perspectives...More than one or two carers should be involved” Kara, 2016, p89*  *"involving families and professionals at this early stage was extremely valuable to the outcome of the study. I feel this ensured that we were on the right path from the start and if there was any uncertainties they were resolved early on." parent, Lakhanpaul et al, 2014, p220* |  |
| Considerations that can come with the caring role | - Time commitments of carers - Emotional and ethical issues around caring | *“realization that the average caregiver in the community was heavily burdened, and would likely have neither the time or the energy to attend regular steering committee meetings" Hagen, 1998, p90*  *“further conversation about the meaning of values would have benefited understanding further… but tried to limit the length of the session due to time limits,” Kowe et al, 2021, p1517*  *“their full, complex ad hectic lives meant they could only be involved in the research where it had direct meaning for them,” Cook et al, 2019, p810*  *“describing their own personal circumstances members found this upsetting as they had to relive their story," Mitchell et al, 2020, p6*  *“some carers were reluctant to talk about the person they cared for as this compromised their privacy; others found it hard to know when to stop talking about their own situation." Repper et al, 2007*, p205 |  |
| Involvement that is efficient, supportive and meaningful for carers | - Important to use carers time efficiently - No one size fits all - Carers design the involvement - Flexibility - Ongoing evaluation of practice - Virtual communication - Multiple carers - Sensitivity and support | *“found the monthly telephone calls an important mechanisms for staying engaged with study progress and assessing how they could add most value to the study, such as offering to interview carers," Lobban et al, 2020, IMPART, p123*  *“Having a clear agenda, however, helped ensure core scheduled items were still returned to and addressed in the course of the meetings. " Grande et al, in press, p10-11*  *“consultations with family carers prior to designing the project,” Cook et al, 2019, p809*  *“the group determined meeting times, location, length of meetings,” Garner & Fauchner, 2014, p67*  *“appreciated the online meetings because they could be at home in case their family members needed them" Elliot, 2013, p123*  *“a key message from the discussion on methods of involvement was that no one size fits all...the optimum strategy was to employ a variety of methods," Banfield et al, 2018, p1226*  *“More than one or two carers should be involved...beneficial...if a carer is unable to attend for any reason” Kara, 2016, p89*  *“give carers an opportunity to tell their story” Grande et al, 2023, p8-9*  *“Personal support from their membership of a group where others understood the pressures of being a carer and supported each other," Mitchell et al, 2020, p6*  *“cognisant of the status of their family situation” Watson, 2016, p96* |  |
| **Carers create change**  *Carers get into research to make a difference, therefore a focus on enabling their involvement in creating change is crucial. Involving the public in health research has been found to lead to service improvement and empower marginalized communities to lead in this change. However, the constraints on change that arise can be sources of frustration for carers.* | | | |
| Carers are passionate about change and can improve services | - Carers motivation to help (which inspires others) - Improves intervention development, effectiveness and implementation - Providing carers with the chance to make a difference | *"to effect change, to be involved in creating something better,” carer, Litherland et al, 2018, p1041*  *“Feeling like our family's experience could be of some help to other families in the future" eparent blog pt II, 2017*  *“Prompted the researcher to go into action,” O’Sullivan et al, 2013, p25*  *"as well as suggestions for how to overcome barriers to putting the intervention into practice; making (DREAMS:START) more accessible and usable for those in need...helped us to develop a complex intervention in an accessible and engaging way" Rapaport et al, 2018, p977*  *“it ended up being a good thing because everyone helped one another with the treatment,” father, Devlin et al, 2022, p729*  *"The process of coding the data and discussing study findings within PPI meetings also enabled PPI groups to contribute to recommendations about how to improve access to therapy" Berry et al 2022 p7* |  |
| Involvement benefits and empowers carers | - Giving carers a voice - Building capacity - Self-development and benefits to wellbeing for carers involved - Strengthens caring capabilities - Improved relationships with health services | *“imparting skills…capacity building, enabling parents to begin leading change in their localities,” Walmsley & Mannan, 2009, p274*  *"Evaluation provides a point of power for families, enabling them to transform their mutual concerns into research questions and data that can then be harnessed to advocate for changes within the system," Painter et al, 2011, p166*  *“for those whose voices were seldom heard," Cook et al, 2019, p808*  *„I felt heard“ carer, RTAG, 2022, p19*  *“I have become a more confident person…the sense of achievement is great,” carer, Kennedy et al, 2011, p10*  *„unexpected gift at the end of the project is that for the first time in my life, I’m loving writing,“ carer, RTAG, p19*  *“working with children in similar circumstances, the children gained confidence and learned new coping skills and strategies from their peers.” Elliot, 2013, p91*  *“photography can be used as a the basis of conversations betweens providers and their patients…starting point to work collaboratively toward family-tailored care,” Postma et al, 2015, p597* |  |
| Constraints on change | - Gap between research and action - Constraints for change limited by resources | *“frustrating waiting for results to be gathered and disseminated, especially when you feel so connected to the research subject. We realised that the speed at which we want to see the results of research shared with the wider public can be at odds with the work the research team has to do. The experience of living with dementia brings an urgency” carer, Litherland et al, 2018, , p1041*  *"challenge was balancing the FTD support group members' desire for advocacy and immediate action to assist others in the community with the very real limitations in professional resources, and the need to carefully evaluate the telehealth support group,” Morgan et al, 2014, p9*  *“Research BUddy was keen to involve other sites and extend recruitment to include patients, which was not feasible for this doctoral study” Coupe & Mathieson, 2020, p131*  *“providers felt they did not have the resources to offer such materials,” Pletch et al, 2015, p167* |  |
| How to help carers make change happen | - Involve key stakeholders - Make carers involvement visible - Long-term planning - Groups of carers build capacity - Training of carers - Communicate the difference they are making | *“Professionals would be a valuable addition…creating a dialogue between parents and service providers,” Walmsley & Mannan, 2009, p275*  *“youth got up there to present, it was meaningful for the adult stakeholders who came to hear directly from them about their experiences," Elliot, 2013, p13*  *“69.2% who attended the photograph exhibit said it was an important and meaningful part,” Levy et al, 2020, p88*  *“advocacy training has been suggested as an important component to photovoice,” Postma et al, 2015, p597*  *“tangible evidence of their contributions were key to their engagement,” McCoy et al, 2019, p11* |  |
